# Matrix Stiffness Governs Fibroblasts’ Regulation of Gingival Immune Homeostasis

**DOI:** 10.1002/adma.202520717

**Published:** 2026-02-08

**Authors:** Hardik Makkar, Nghi Tran, Yu‐Chang Chen, Kang I. Ko, Rebecca G. Wells, Kyle H. Vining

**Affiliations:** ^1^ Center For Innovation & Precision Dentistry University of Pennsylvania Philadelphia USA; ^2^ Department of Preventive and Restorative Sciences School of Dental Medicine University of Pennsylvania Philadelphia USA; ^3^ Department of Bioengineering School of Engineering and Applied Sciences University of Pennsylvania Philadelphia USA; ^4^ Department of Materials Science and Engineering School of Engineering and Applied Sciences University of Pennsylvania Philadelphia USA; ^5^ Department of Periodontics School of Dental Medicine University of Pennsylvania Philadelphia USA; ^6^ Center For Engineering Mechanobiology University of Pennsylvania Philadelphia USA; ^7^ Department of Medicine Perelman School of Medicine University of Pennsylvania Philadelphia USA

**Keywords:** fibroblast–immune crosstalk, gingival extracellular matrix, matrix stiffness, mechanotransduction, nuclear organization, periodontal disease

## Abstract

Periodontal disease is characterized by inflamed gingival tissues and degradation of the gingival extracellular matrix (ECM), yet the role of mechanical cues remains poorly understood. Gingival ECM in periodontal disease showed reduced fibrillar collagen compared to healthy samples. We hypothesized that ECM softening in periodontal disease contributes to inflammation by dysregulating gingival fibroblasts (GFs). A mechanically tunable hydrogel model of the gingival ECM was developed to investigate the mechano‐immune crosstalk. Stiff and soft collagen‐alginate hydrogels matched the rheological properties of healthy and diseased gingival biopsies respectively. Human donor GFs encapsulated in these stiff hydrogels showed significantly suppressed toll‐like receptor‐mediated inflammatory responses compared to those in soft hydrogels. The non‐canonical NFκB pathway and epigenetic nuclear organization directed stiffness‐dependent inflammatory responses of GFs. The direct impact of mechanical cues on immune responses was investigated ex vivo by co‐culture of donor‐derived human GFs with myeloid cells and in human gingival explants. Myeloid progenitors co‐cultured with GFs in stiff hydrogels differentiated into immunomodulatory dendritic cells. Ex vivo crosslinking of human gingival tissue increased stiffness and reduced the production of inflammatory cytokines. Gingival mechano‐immune regulation offers a novel approach to biomaterial‐based treatments for periodontitis.

## Graphical abstract

This study identifies a “mechano‐epigenetic” checkpoint in which matrix stiffness governs the immune responses of gingival fibroblasts. Using biomimetic hydrogels and human gingival explants, the studies revealed that restoring extracellular matrix stiffness actively suppresses inflammation via non‐canonical NF‐κB signaling and DNA methylation, offering a novel therapeutic paradigm.

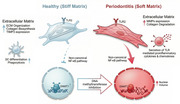

## 1 Introduction

Severe periodontitis affects 19% of adults globally, causing tooth loss and increasing the risk of systemic diseases like heart disease, diabetes, respiratory infections, and adverse pregnancy outcomes [1, 2, 3]. The World Health Organization has prioritized an action plan for this condition due to its significant impact on health, quality of life, and healthcare costs, which exceed $40 billion USD. The primary focus on periodontal disease pathogenesis has been on microbial dysbiosis and its resulting hyper‐inflammatory host response [2, 4, 5, 6]. The contribution of extracellular matrix (ECM) mechanical properties has been extensively explored in other chronic inflammatory diseases, such as rheumatoid arthritis and musculoskeletal injuries [7]. However, its contribution to gingival tissues remains understudied, even though host and microbial proteases expressed in periodontitis may significantly impact the gingival ECM [2, 8, 9]. There remains a knowledge gap regarding how the physical microenvironment of the gingival connective tissue and its ECM regulates immune homeostasis.

Gingival fibroblasts (GFs) are the resident cell type of the gingival connective tissue. GFs play a crucial role in regulating the mechano‐immune axis [10, 11, 12, 13, 14]. They are essential to both tissue homeostasis and the pathogenesis of periodontal disease [15, 16, 17, 18, 19]. GFs act as key sentinel cells in the immune response against pathogen‐associated molecular patterns (PAMPs), mainly through Toll‐like receptor 2 (TLR2) signaling [6, 10, 13, 20, 21, 22]. We propose that ECM breakdown and tissue softening in periodontitis triggers a switch in GF phenotype from quiescent, tissue‐building cells into pro‐inflammatory, matrix‐degrading effectors that enhance, rather than dampen, inflammation. This establishes a destructive feedback loop in which matrix breakdown triggers a pathological immune response, which in turn degrades more matrix and further softens the surrounding tissue [10, 11, 12, 14, 23]. These mechanical cues have the potential to impact the nucleus (epigenome and genome) and the transcriptional control of immune responses [23].

We investigated the mechanobiology of GFs using a tunable 3D gingival ECM‐mimicking hydrogel system [24, 25, 26, 27] composed of interpenetrating networks of collagen and alginate biopolymers. This composite system combines a bioinert, ionically cross‐linked alginate network that allows independent control of stiffness without altering ligand density or diffusivity with type I collagen, the primary extracellular component of gingival connective tissue. The resulting matrix offers controllable mechanical tunability and native cell‐adhesive cues, enabling a systematic analysis of how defined changes in stiffness influence GF mechanotransduction and inflammatory signaling. Using this 3D platform, we determined how matrix stiffness regulates the GF inflammatory responses to TLR signaling and identified the governing cellular and epigenetic factors. Further, we investigated the integrated impact of mechanical cues on immune homeostasis with stromal‐myeloid co‐culture models. We validated our findings by restoring tissue stiffness through the enzymatic crosslinking of human gingival explants. This approach demonstrated that gingival ECM stiffness is a key regulator of periodontal inflammation, providing a rationale for developing biomaterial‐based treatments to restore gingival health [28].

## 2 Results and Discussion

### 2.1 Collagen Fiber Organization in the Gingival Connective Tissue

**FIGURE 1:**
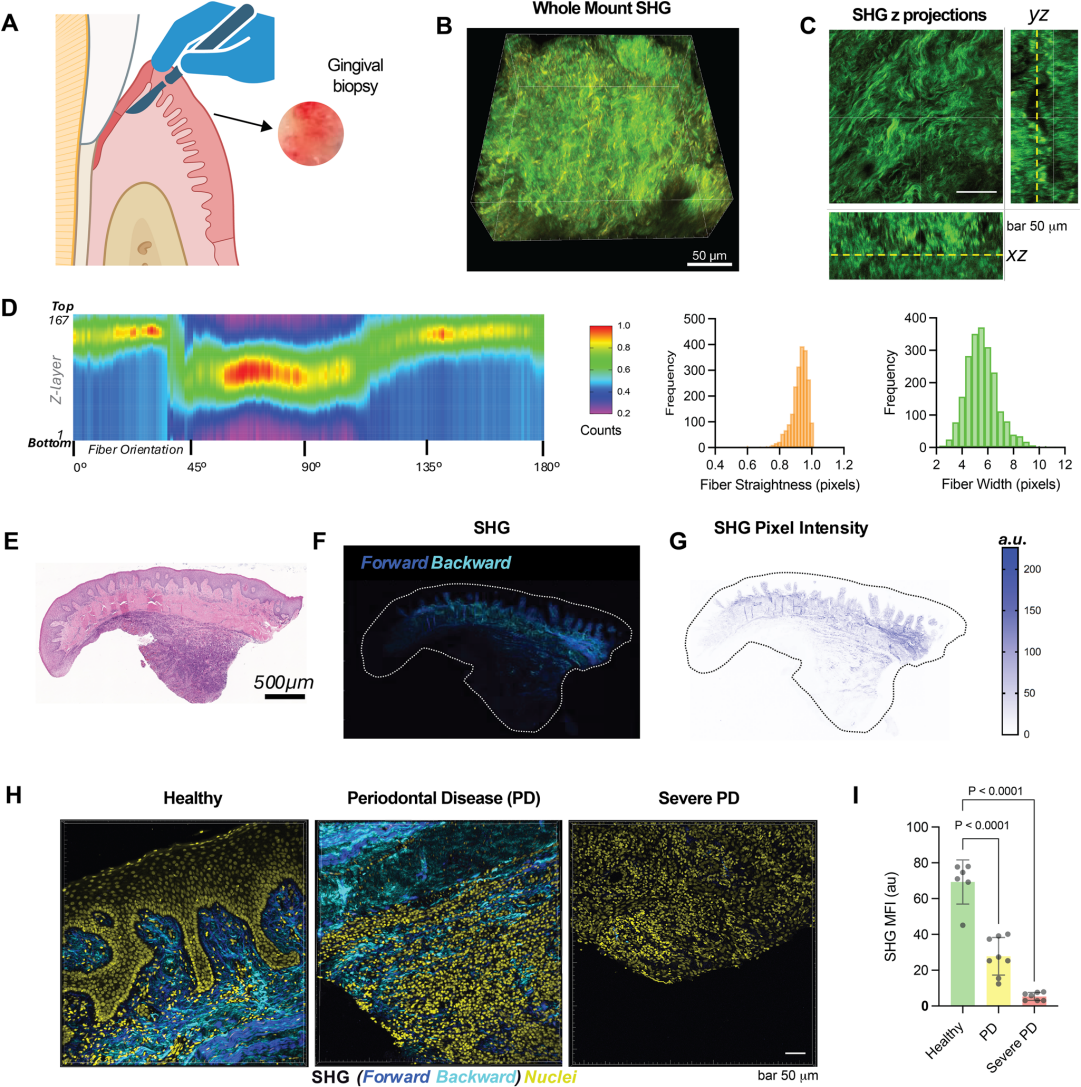
Periodontitis is characterized by a progressive loss of fibrillar collagen organization and density in the human gingival extracellular matrix (**ECM**). (A) Schematic illustrating the experimental workflow for analyzing human gingival ECM. The workflow involves the acquisition of a human gingival tissue biopsy followed by whole‐mount Second Harmonic Generation (SHG) imaging for high‐resolution, label‐free analysis of collagen architecture. Created with BioRender.com. (B) Representative whole−mount SHG image of a healthy gingival connective tissue sample. Collagen fibers (green) are clearly visible. (C) SHG maximum z−projection images (*XY*, *YZ*, and *XZ* planes) demonstrating the organization of collagen fibers through the tissue depth. (D) Heatmap showing the distribution of collagen fiber orientation as a function of Z−layer depth, corresponding histograms quantify fiber straightness and width. (E) Hematoxylin and Eosin (H&E) staining of a representative human gingival tissue section. (F) SHG forward and backward signal imaging of the tissue section outlined in (E), revealing the collagen distribution. (G) SHG pixel intensity map across the section in (F), providing a quantified spatial representation of collagen content. (H) Representative SHG micrographs of gingival connective tissue from Healthy, Periodontal Disease (PD), and Severe PD sites. Images show merged signals of forward‐scattered SHG (blue), backward‐scattered SHG (cyan), and nuclei (yellow). (I) Quantification of the collagen density by measuring the Mean Fluorescence Intensity (MFI) of the SHG signal across the three disease stages. Data are shown as mean ± SD (n = 3 biologically independent donors; dots indicate individual technical replicates derived from these donors. *p*‐values were determined by ordinary one‐way ANOVA followed by Dunnett's multiple comparisons test and are indicated on the graph.

Whole‐mount Second Harmonic Generation (SHG) confocal microscopy of healthy gingival connective tissue was used to examine collagen fiber orientation and structure (Figure 1A–D; Figure S1A). The heatmap illustrates the distribution of collagen fiber pixel percentage counts at various angles (*x*‐axis) and Z‐layers (*y*‐axis), with color intensity indicating the count (red for the highest counts, pink for the lowest) (Figure 1D). In the middle Z‐layers, collagen fibers were oriented between 45°–90° (Figure 1D), suggesting organized fibers within the bulk of the connective tissue [29, 30]. In contrast, fibers in the superficial and deep Z‐layers (toward the top and bottom of the heatmap) were oriented between 0°–45° and 135°–180°. This organization, characterized by a high‐density band of fibers in the middle Z‐layers, surrounded by regions of lower density, is consistent with a stratified arrangement of collagen fibers in the gingival tissue [29, 31] and reflects the mechanical and structural requirements of the gingival tissue [32].

### 2.2 Periodontal Disease is Linked With Pathological Changes to the Extracellular Matrix

Experimental models and human biopsies consistently demonstrate a significant reduction in collagen area and altered fibril architecture during disease [31]. Periodontal pathogens and their secreted cysteine proteases not only degrade collagen directly but also induce gingival fibroblasts and epithelial cells to upregulate matrix metalloproteinases (MMP‐1, MMP‐2, MMP‐3, MMP‐9) while suppressing their inhibitors (TIMPs), amplifying host‐mediated collagen degradation [33]. Enzymatic breakdown and an imbalance between MMPs and TIMPs lead to a weakened, disorganized ECM [34, 35, 36]. Multimodal imaging and quantitative analysis were performed to investigate changes in collagen fiber organization in diseased gingival connective tissue compared to healthy controls (Figure 1E,F). First, we examined SHG images of tissue sections from healthy sites (Figure 1F–H; Figure S1A,B), which showed a robust, orderly collagen network with little to no apparent immune cell infiltration, consistent with a lack of inflammation. Collagen fibers in sections of healthy tissue exhibited a bimodal distribution in the orientation histogram with two major peaks at ±50°−55° (Figure S1C,D). In contrast, SHG images of diseased regions revealed a significant decrease in the coherent SHG signal, which appeared fragmented, indicating collagen degradation and disorganization (Figure 1F–I; Figure S1B–D). These images also showed heavy immune cell infiltration. Quantitative angular analysis of diseased tissue (Figure S1C,D) showed a less organized collagen fiber distribution with a greater proportion of randomly oriented fibers over a wider range of angles (Figure S1D). This diffuse distribution is consistent with large‐scale collagen degradation and the infiltration of inflammatory cells observed in the microscopy images. These imaging results align with prior histological and molecular studies demonstrating that periodontal inflammation drives extensive collagen degradation and remodeling of the gingival ECM [35].

### 2.3 Gingival ECM Hydrogel Models the Rheological Properties of Human Gingival Tissue

**FIGURE 2:**
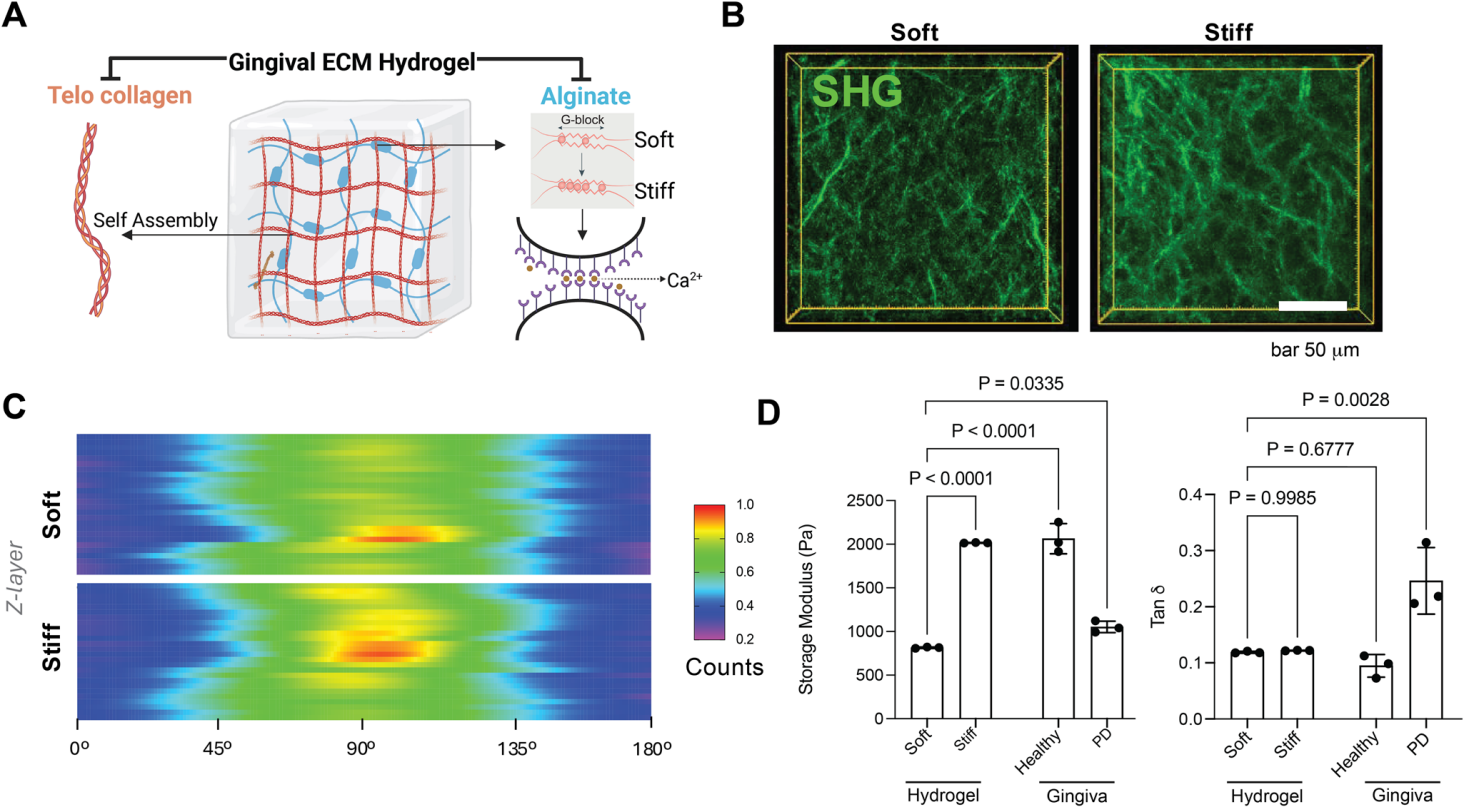
A biomimetic alginate‐collagen interpenetrating hydrogel system recapitulates the tunable stiffness and viscoelasticity of human gingival **ECM**. (A) Schematic illustrating the design of the interpenetrating polymer network (IPN) hydrogel system. It is composed of self‐assembled type I collagen (telo collagen) and ionically cross‐linked alginate (via Ca2+ ions interacting with G‐blocks). Created with BioRender.com. (B) Representative Second Harmonic Generation (SHG) images showcase the fibrillar collagen architecture within the soft and stiff hydrogel formulations. (C) Heatmap analysis of collagen fiber orientation distribution throughout the z−depth of the soft and stiff hydrogels. (D) Rheological characterization of the developed hydrogels compared to native healthy and diseased (PD stage 2 and 3) human gingival tissue. Bar graphs show the Storage Modulus (G′) and Tan Delta (Tanδ) (ratio of loss modulus G′′ to G′), representing viscoelasticity. Data are presented as mean ± SD. Sample size n = 3 biologically independent donors per group for all rheology measurements. P‐values were determined by ordinary one‐way ANOVA followed by Dunnett's multiple comparisons test and are indicated on the graph. Scale bars are as indicated.

We developed gingival ECM hydrogels using a collagen‐alginate interpenetrating network hydrogel system with tunable rheological properties to model the ECM microenvironments of healthy and diseased gingival tissue [24, 25, 27]. Ionic cross‐linking of the alginate network with calcium carbonate provided tunable storage moduli (G′) while maintaining a constant concentration of bovine Type I telo‐collagen (4 mg/mL) and ultra‐pure low molecular weight alginate (1 wt.%). SHG heatmaps of the hydrogels mimicked collagen organization found in healthy gingival tissue (Figure 2A–C), with a band of collagen fibers orientated between 45–135 degrees, similar to the central high‐density zone of native gingiva (Figures 1D and 2C). Shear stress relaxation tests showed the viscoelastic behavior of the ionically cross‐linked hydrogels (Figure S2A,B). The stiff and soft ECM hydrogels matched the rheological properties of healthy and diseased gingiva, respectively (Figure 2D; Figure S2C). The G' of gingiva was significantly reduced from ∼2000 Pa in healthy sites to ∼1000 Pa in periodontal disease (Stage 2 and 3). The reduction in G’ is likely attributable to the degradation of the collagen network and other ECM components [3, 14, 34]. The diseased gingiva also exhibited a significant increase in the loss tangent (tan δ) compared to healthy controls, indicating a more dissipative, fluid‐like material [25, 26]. The G’ of the “Soft” hydrogel was slightly lower than that of diseased tissues (Figure 2D). Overall, these rheological measurements demonstrate that tunable hydrogels can match the range of mechanical properties of healthy (stiff) and diseased (soft) gingival tissue.

### 2.4 Stiff ECM Hydrogels Upregulate Matrix‐Related Pathways in Gingival Fibroblasts

**FIGURE 3:**
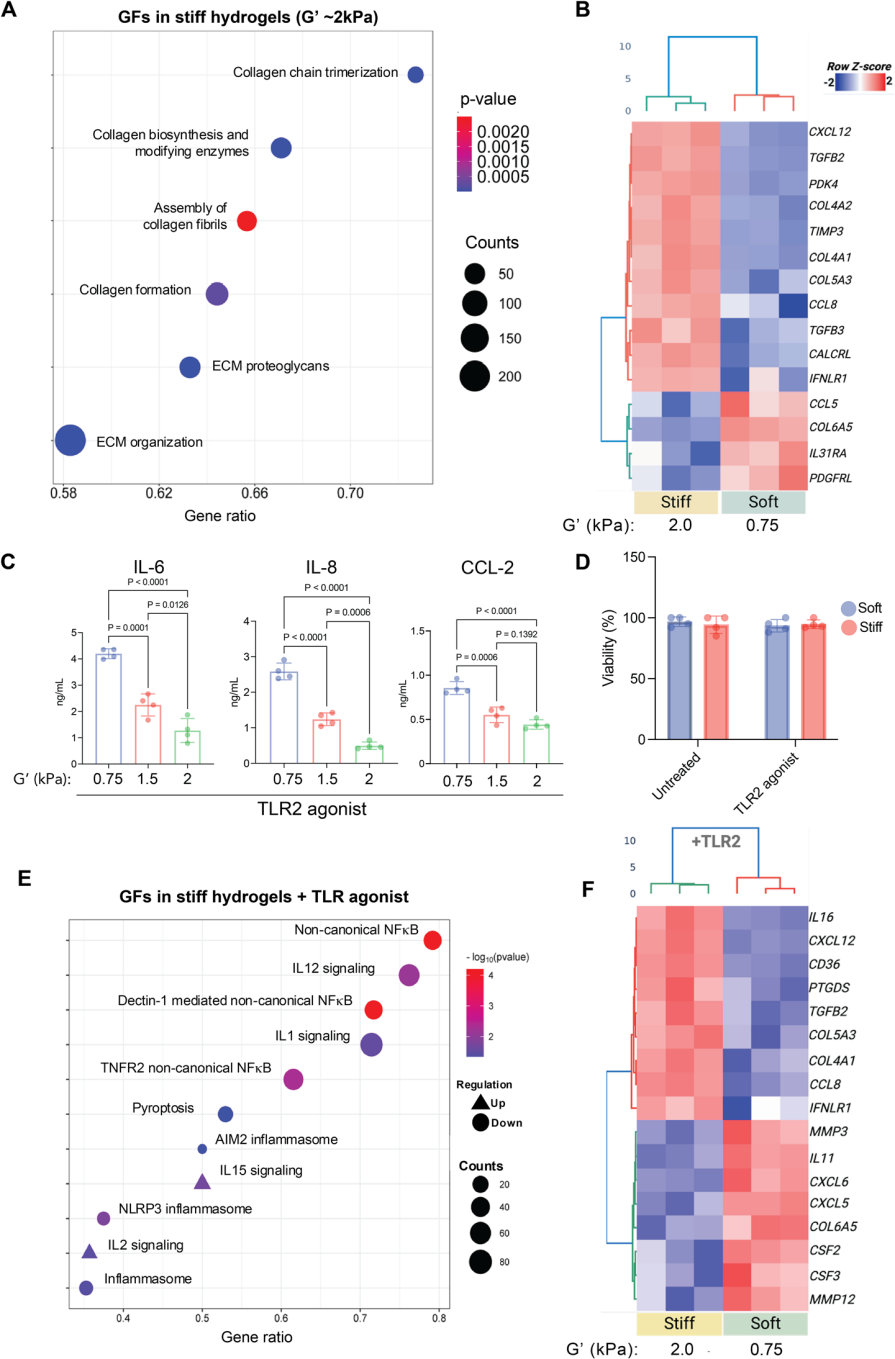
Matrix stiffness drives a pro‐anabolic phenotype, while reduced stiffness promotes TLR2‐mediated inflammatory responses in human GFs. (A) Bubble plot showing the gene set enrichment of pathways significantly upregulated in GFs cultured on stiff hydrogels (G′ approx 2 kPa) compared to soft hydrogels (G′ approx 0.75 kPa) (n = 3). Pathways are predominantly related to the production and organization of ECM. The gene ratio (*x*‐axis) is the proportion of differentially expressed genes in a pathway. Bubble size indicates the count (number of genes in the pathway), and the color scale represents the Benjamini–Hochberg corrected *p*‐value. (B) Heatmap illustrating the relative expression of differentially expressed genes (z‐scored) in the stiff versus soft hydrogel environments (n = 3). (C) ELISA quantification of secreted pro‐inflammatory mediators IL‐6, IL‐8, and CCL‐2 from GFs cultured in hydrogels of varying stiffness (G′: 0.75, 1.5, and 2 kPa) following TLR2 activation (n = 4). Data are presented as mean ± SD. *p*‐values were determined by ordinary one‐way ANOVA followed by Tukey's multiple comparisons test. (D) Cell viability assay showing that neither hydrogel stiffness nor TLR2 agonist stimulation significantly impacted GF viability over the culture period (n = 4). Data are presented as mean ± SD. (E) Bubble plot showing the gene set enrichment of inflammatory signaling pathways regulated in GFs cultured in stiff hydrogels compared to those cultured in soft hydrogels, both with TLR2 agonist stimulation (n = 3). Bubble size corresponds to the count, and color scale indicates the −log10(*p*−value) of the pathway. Circles indicate downregulation, and triangles indicate upregulation in stiff hydrogels. (F) Heatmap showing the relative expression of differentially expressed genes (z‐scored) in GFs cultured in soft versus stiff hydrogels following TLR2 agonist stimulation (n = 3). For all panels, *p*‐values <0.05 were considered statistically significant and are indicated on the graphs.

Gingival fibroblasts (GFs) were isolated from healthy donor tissue. Flow cytometry showed GFs were positive for markers of mesenchymal stromal cells (MSCs), including CD105, CD73, CD90, and CD44 (Figure S2D). Next, GFs were encapsulated in soft and stiff gingival ECM hydrogels. Pathway enrichment analysis of upregulated genes from bulk RNA sequencing data identified enrichment for ECM synthesis and organization. Transcriptional programs related to collagen synthesis and maturation were enriched in stiff hydrogels (Figure 3A), including the Assembly of Collagen Fibrils, Collagen biosynthesis and modifying enzymes, Collagen formation, and Collagen chain trimerization. ECM organization was the pathway with the largest number of associated genes (200 counts). Taken together, these findings illustrate that the mechanical stiffness of the 3D matrix promotes fibroblasts to adopt an active, matrix‐synthesizing phenotype, which may help preserve tissue integrity (Figure 3A). In stiff hydrogels, GFs also upregulated genes reflective of a homeostatic and matrix‐maintaining phenotype, typical of healthy gingival connective tissue, such as COL5A3, COL4A2, COL6A5, and COL4A1 (Figure 3B; Figure S3A). The upregulated expression of TIMP3, a matrix metalloproteinase inhibitor, in conjunction with TGFB2 and TGFB3, further supports a transcriptional program of ECM deposition, maturation, and resistance to degradation. Metabolic and signaling genes, such as PDK4 and CALCRL, were also upregulated in GFs cultured in stiff hydrogels (Figure S3A). These results were consistent with the organized collagen architecture and strong mechanical properties of native healthy gingiva [37].

### 2.5 TLR Mediates Inflammatory Responses in Gingival ECM Hydrogels

We investigated how the ECM mechanical properties modulate the inflammatory response of gingival fibroblasts (GFs) to microbial mimetics. The Toll‐like receptor 2 (TLR2) in gingival fibroblasts recognizes a broad spectrum of bacterial components, especially those from abundant Gram‐positive and Gram‐negative bacteria in periodontal biofilms [14, 18, 22, 38]. TLR2 exhibits a broad recognition profile relevant to the initiation and maintenance of chronic inflammatory conditions in gingival fibroblasts [14], whereas TLR4 recognizes Gram‐negative bacterial lipopolysaccharide (LPS) [39]. Soft and stiff gingival ECM hydrogels were challenged with a TLR2 agonist to determine whether stiffness regulates fibroblast inflammatory responses. Gene expression of the TLR2 pathway was not significantly modulated by matrix stiffness, except for the downregulation of CD14 (Figure S3C). ELISA analysis demonstrated a dramatic, stiffness‐dependent reduction of secreted pro‐inflammatory cytokines. Fibroblasts within soft 3D matrices secreted increased amounts of the cytokine IL−6 and the chemokines IL−8 (CXCL8) and CCL2 (MCP‐1) in response to TLR2 stimulation (Figure 3C; Figure S4A). In contrast, increasing the stiffness of the hydrogel resulted in a progressive decline in TLR2‐mediated secretion of pro‐inflammatory cytokines. Fibroblasts cultured in the stiffest (2 kPa) 3D matrices produced the lowest amount of IL−6, IL−8, and CCL2. Simulating other innate immune pathways, with agonists for Toll‐like receptors 3 (TLR3) and 4 (TLR4), showed a similar pattern. GFs in soft hydrogels responded to TLR3 and TLR4 agonists with IL‐6 and IL‐8 expression (Figure S4B), which was significantly dampened in stiff hydrogels. For simplicity, the remaining studies focused on TLR2‐mediated inflammation. Overall, these results demonstrate that increased ECM stiffness suppressed the inflammatory response of GFs to mimicked microbial challenge, suggesting that 3D mechanotransduction is a crucial regulator of the fibroblast inflammatory secretome [40, 41].

Results from bulk RNA‐seq analysis indicate that GFs in soft hydrogels exhibit upregulation of genes commonly associated with inflammation, immune cell recruitment, and tissue breakdown, mirroring the pathologic alterations observed in diseased gingiva. Chemokines CXCL12, CSF3, CCL8, CXCL6, and CXCL5 were highly upregulated in soft gels, indicating strong chemoattractant signaling for immune cells, characteristic of periodontal inflammation (Figure 3E,F; Figure S3B). Additionally, matrix metalloproteinase genes MMP3 and MMP12, as well as disintegrin and metalloproteinase with thrombospondin motifs ADAMTS14, were upregulated in soft gels, indicating ongoing ECM breakdown [14]. Overall, data suggest stiff hydrogels promote a gene expression profile that actively maintains ECM integrity and regulates inflammation. Conversely, soft hydrogels mirror the disease state by promoting a strong pro‐inflammatory and matrix degradative gene signature.

### 2.6 Gingival Fibroblasts Exhibit Morphological Responses to Matrix Stiffness

**FIGURE 4:**
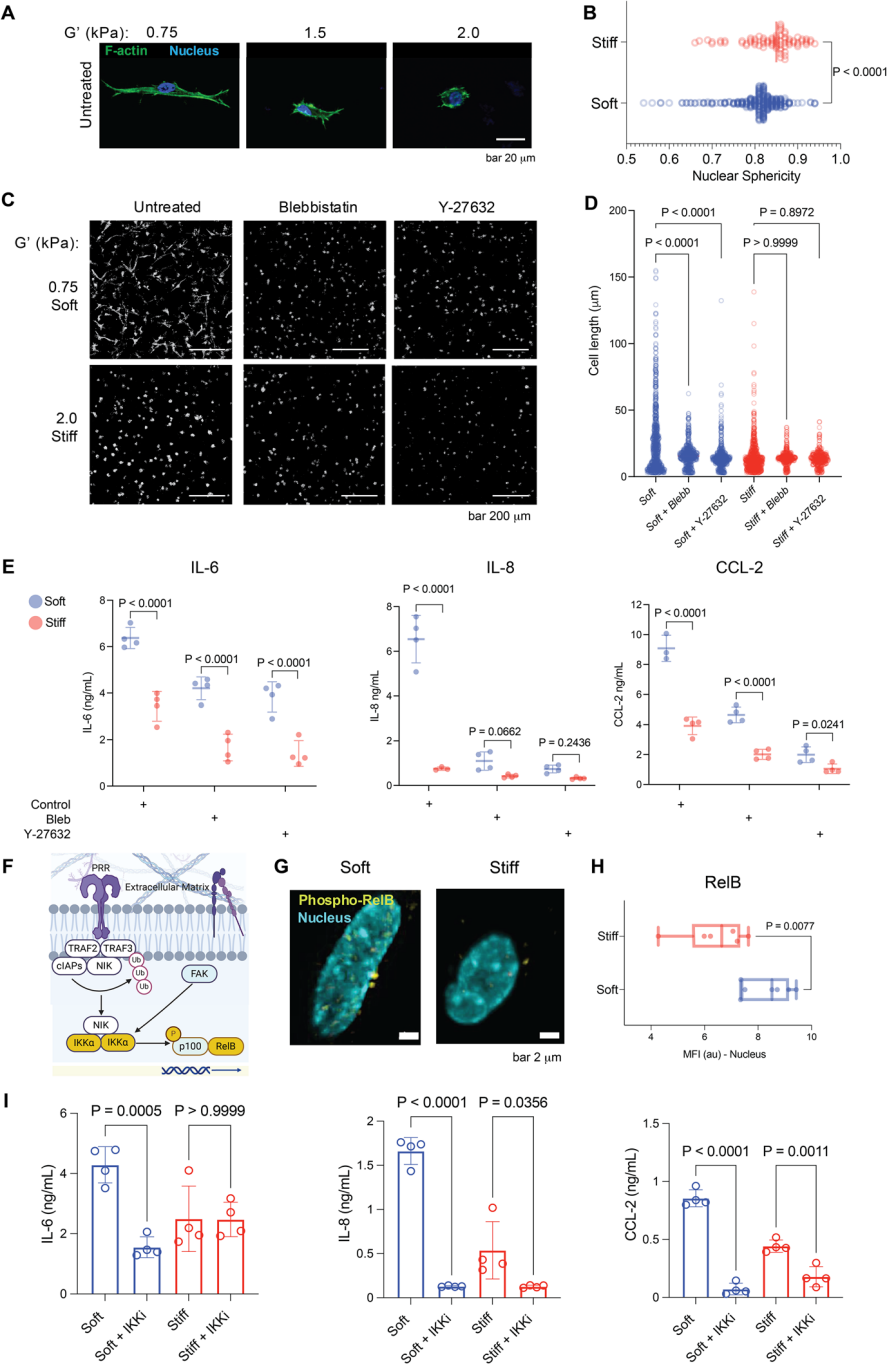
Cytoskeletal tension and the non‐canonical **NF−κB** pathway regulate matrix stiffness‐dependent inflammatory responses in gingival fibroblasts (GFs). (A) Representative confocal immunofluorescence images showing F‐actin organization (Phalloidin, green) and nuclear morphology (DAPI, blue) in GFs cultured on hydrogels with increasing stiffness (G' approx 0.75, 1.5, and 2.0 kPa). (B) Quantification of nuclear sphericity. Dots represent individual nuclei from three independent experiments. n = 319 (soft) and n = 81 (stiff). *p* values were determined by a two‐tailed unpaired t‐test. (C) Representative images and (D) quantification of cell length in GFs following treatment with the non‐muscle myosin II inhibitor Blebbistatin (Blebb) or the ROCK inhibitor Y‐27632. Dots represent individual cells. n = 787 (soft), n = 539 (soft+blebb), n = 539 (soft+Y27632), n = 688 (stiff), n = 359 (stiff+ blebb), n = 358 (stiff+Y27632) pooled from three independent experiments. *p* values were determined by two‐way ANOVA followed by Šídák's multiple comparisons test. (E) ELISA quantification of IL‐6, IL‐8, and CCL‐2 secretion from TLR2‐activated GFs treated with cytoskeletal inhibitors (n = 4) *p* values were determined by two‐way ANOVA followed by the original FDR method of Benjamini and Hochberg. (F) Schematic representation of the proposed mechanism: ECM stiffness and TLR2 signaling converge to regulate non‐canonical NF‐κB signaling in GFs. Created with BioRender.com. (G) Representative immunofluorescence images, and (H) quantification of the Mean Fluorescence Intensity (MFI) of nuclear‐localized phosphorylated RelB (Ser552) in cells cultured in soft versus stiff hydrogels (n = 3) *p* values were determined by a two‐tailed unpaired t‐test. (I) ELISA quantification of IL‐6, IL‐8, and CCL‐2 secretion from TLR2‐activated GFs cultured in soft or stiff matrices with or without IKK inhibition (n = 4). *p* values were determined by two‐way ANOVA followed by Šídák's multiple comparisons test. Scale bars are as indicated. Data are presented as mean ± SD, and specific *p*‐values are indicated directly on the graphs, where *p*<0.05 was considered statistically significant.

In soft ECM hydrogels, the gingival fibroblasts displayed a characteristic, highly spread morphology with pronounced uniaxial protrusions (Figure 4A; Figure S4C). These cellular extensions were often seen to be tipped by lamellipodial blebs, reflecting active membrane dynamics underpinned by actin polymerization and cytoskeleton‐mediated probing [24]. The F‐actin cytoskeleton (green) was loosely organized into discrete stress fibers that extended into the processes of the cell, reflecting an active probing nature (Figure 4A–C), consistent with its role in migration and remodeling [42, 43]. The cell body, although spread, remained relatively elongated, and the nucleus (blue) was distended, in agreement with cells probing a compliant environment [24]. In contrast, the spreading of gingival fibroblasts in stiff hydrogels was significantly hindered. The cells displayed a more compact, round, or stellate morphology. The formation of uniaxial extensions and lamellipodial blebs was significantly reduced. While F‐actin (green) was still visible, it was organized into a more compact and highly tensed structure within the limited cellular space (Figure 4A). The nucleus also showed a more spherical morphology compared to cells in softer gels, reflecting the altered cytoskeletal tension and overall cellular confinement without a reduction in viability (Figures 4B and 3D). The compact morphology in stiff gels is indicative of cell‐matrix signaling [44].

### 2.7 Actomyosin Contractility Determines Fibroblast Morphology and Inflammatory Response in a Stiffness‐Dependent Manner

GFs encapsulated in soft and stiff hydrogels were exposed to blebbistatin (a myosin II inhibitor) or Y27632 (an inhibitor of Rho‐associated protein kinase (ROCK)), both key disruptors of actin cytoskeleton‐generated forces [24]. Confocal micrographs in Figure 4C characterized the morphological responses to matrix stiffness. In soft ECM hydrogels, inhibition of actomyosin contractility significantly decreased cell length, with the population of fibroblasts showing a shorter, more rounded morphology (Figure 4D). This result is consistent with previous works, which show that the elongated phenotype of GFs in a soft environment is dependent on actomyosin contractile forces [12, 45, 46]. Although GFs were already more compact in stiff hydrogels, inhibition of contractility also caused a further reduction in cell length. Next, GFs in both soft and stiff hydrogels were challenged with a TLR2 agonist and treated with blebbistatin and Y27632, resulting in a dampened inflammatory response across both stiffnesses (Figure 4E). These results suggest that actomyosin contractility is required for GFs to sense ECM stiffness [24, 39, 47] and regulate their inflammatory responses [46, 48].

### 2.8 Mechanotransduction‐Induced Inflammatory Response in GFs is Controlled by Non‐Canonical NF‐κB Signaling

Pathway enrichment analysis was conducted on differentially expressed genes from bulk RNA‐sequencing of GFs activated with a TLR2 agonist in stiff versus soft hydrogels. The bubble plot reveals differentially regulated signaling pathways with the highest fold enrichment (Figure 3E). The most striking result was the strong downregulation of non‐canonical NF‐κB in stiff hydrogels (Figures 3E and 4F; Figure S5A). The analysis also indicated downregulation of multiple inflammasome‐related pathways. Several other inflammasome‐related pathways were upregulated, albeit with generally lower statistical significance than the highly downregulated pathways. These included IL‐15 signaling and IL‐2 signaling. The engagement of these cytokine‐signaling pathways, however modest, may be a compensatory or regulatory response in the healthy‐mimicking matrix, modulating overall immune regulation [49]. To further confirm the downregulation of non‐canonical NF‐κB signaling, we examined the protein levels of RelB, an important transcription factor in this pathway [50]. Immunofluorescence staining and subsequent quantification showed lower RelB mean fluorescence intensity (MFI) in GFs encapsulated in stiff hydrogels (Figure 4G,H).

IKK, a kinase upstream of RelB, was inhibited following TLR2 activation of GFs in hydrogels. The secretion of IL‐6, IL‐8, and CCL‐2 in soft hydrogels was greatly diminished, reaching levels comparable to or even below those of stiff gels. This drastic decrease shows that the activated inflammatory response in soft matrices is reliant on the activity of the IKK complex [50]. In contrast, TLR2‐stimulated GFs in stiff hydrogels had dramatically lower baseline levels of these inflammatory markers, which align with our observations of dampened inflammatory signaling in a healthy mechanical environment. The IKK inhibitor had little to no further effect on IL‐6 expression in stiff hydrogels (Figure 4I). These results are consistent with our hypothesis that the stiff matrix intrinsically downregulates the non‐canonical NF‐κB pathway, making it less responsive to further pharmacological inhibition. Collectively, these functional experiments provide strong evidence that the mechanical regulation of the non‐canonical NF‐κB [50] signaling pathway mediates GF's inflammatory response to mechanical cues in a 3D environment. Previous work showed that GFs cultured on 2D soft and stiff substrates were regulated by a YAP‐canonical NF‐κB axis [12]. This present study advances this concept by demonstrating that the non‐canonical NF‐κB pathway plays a major role in the 3D gingival ECM context.

### 2.9 GFs Exhibit Stiffness‐Dependent Decrease in Overall Nuclear Volume and Transcriptional Activation of Pathways Linked With the ECM‐Cytoskeletal‐Nuclear Axis

Next, we examined the impact of matrix stiffness on nuclear shape. Confocal microscopy revealed that GFs in soft hydrogels had more elongated nuclei, while nuclear sphericity was increased in GFs in stiff matrices (Figure 4B). This result is consistent with the effect of increased cytoskeletal tension, which is transmitted to a compact and spherical nuclear shape [51]. Conversely, the decreased tension in softer matrices allows for a more elongated or irregular nuclear morphology [52]. The nucleus integrates mechanical cues into chromatin structure and gene expression [51, 52, 53, 54]. To further understand this process, we conducted a targeted pathway enrichment analysis of nuclear mechanotransduction of TLR2‐activated GFs in stiff gingival ECM hydrogels (Figure S5B). Integrin cell surface interactions and non‐integrin membrane‐ECM interactions pathways were prominently upregulated, highlighting the crucial roles played by both direct and indirect cell‐ECM adhesion in sensing matrix stiffness. Additionally, Lamin interactions were upregulated, highlighting the physical linkage between the cytoskeleton and the nuclear lamina, which is critical for transmitting physical forces to the nucleus and influencing nuclear shape and transcriptional activity [55, 56]. Immunostaining for Lamin A/C of GFs in soft hydrogels showed a peripheral Lamin A/C distribution, concentrated mainly at the nuclear rim (Figure S5C). This peripheral localization is typically associated with a mechanically rigid nuclear lamina, reduced nuclear deformability, and constrained chromatin organization [51, 52, 56, 57]. In contrast, in stiff hydrogels, Lamin A/C was more diffusely distributed throughout the nucleoplasm, suggesting a more deformable nucleus that could enhance the transmission of mechanical signals to chromatin [51, 52].

Next, we examined the role of actomyosin contractility on nuclear morphology. GFs in stiff gingival hydrogels consistently showed a much smaller nuclear volume than GFs in soft gels, regardless of the presence of inhibitors of actomyosin contractility (Figure S5D). Notably, nuclear volume in inhibitor‐treated stiff gels was still much lower than in inhibitor‐treated soft gels. Extracellular matrix stiffness largely determines the baseline nuclear volume, likely through external mechanical confinement and/or stiffness‐mediated influences on nuclear chromatin density [57]. These results are consistent with previous works showing that active cellular actomyosin contractility acts as a dynamic modulator of nuclear volume and shape [46, 52]. We propose that the nucleus is regulated by both ECM stiffness and its epigenetic landscape [54] in periodontal health and disease.

### 2.10 Inhibition of DNA Methyltransferase Eliminates the Effect of Matrix Stiffness on Nuclear Morphology, Spreading, and Inflammatory Response of Gingival Fibroblasts

**FIGURE 5:**
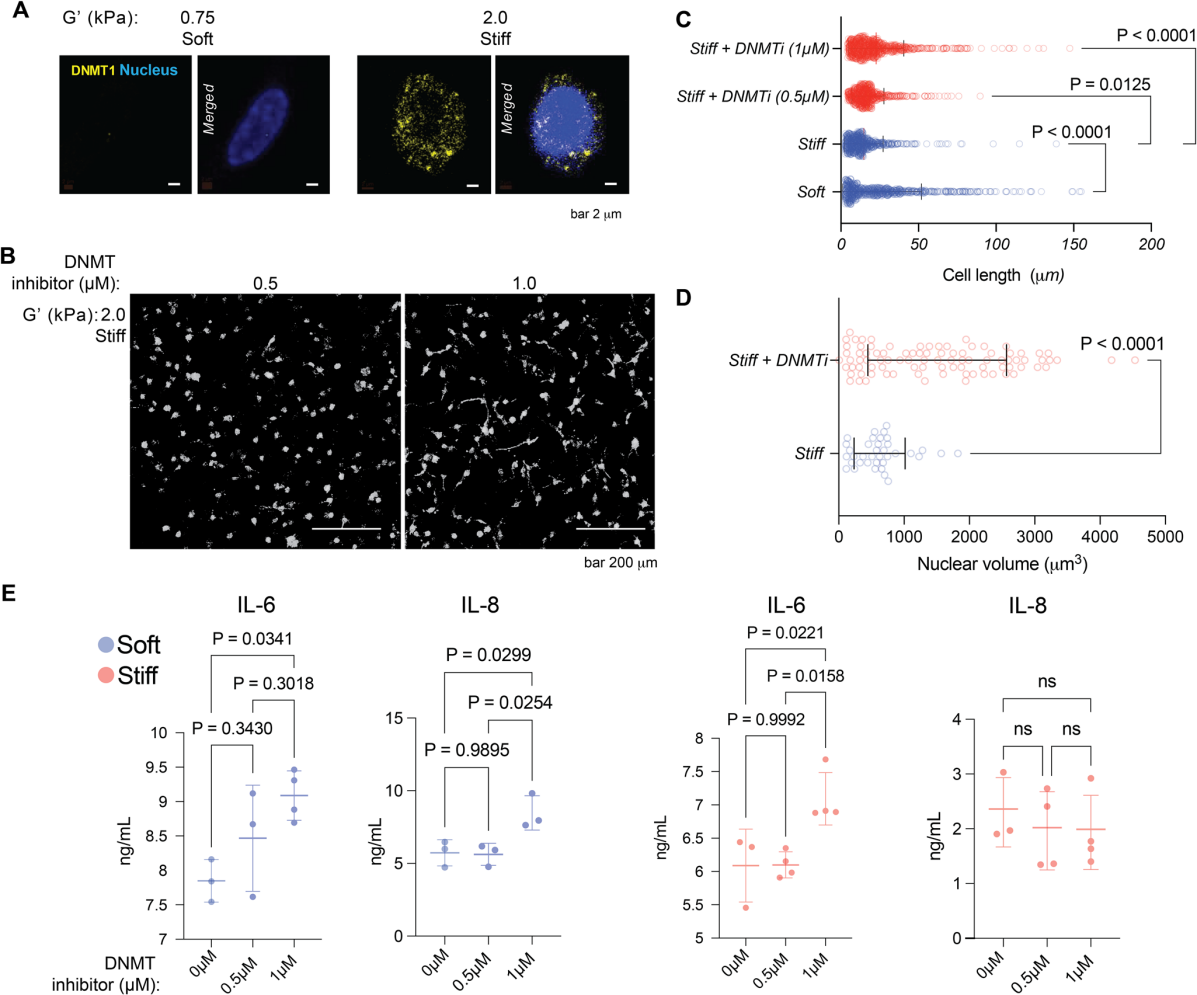
DNMT1 inhibition rescues the mechanical phenotype and restores pro‐inflammatory cytokine secretion by fibroblasts in stiff matrices. (A) Representative confocal immunofluorescence images showing the localization of DNMT1 (yellow) and the nucleus (DAPI, blue) in gingival fibroblasts (GFs) cultured in soft (G' 0.75 kPa) versus stiff (G' 2.0 kPa) ECM hydrogels. (B) Representative *z*‐projection images of phalloidin‐stained GFs encapsulated in stiff hydrogels (G' 2.0 kPa) treated with increasing concentrations of the DNMT inhibitor (0.5 and 1.0 µM). (C) Quantification of cell length. Dots represent individual cells. n = 787 (soft), n = 688 (stiff), n = 710 (stiff+DNMTi 0.5 uM) and n = 917 (stiff+DNMTi 1 uM) pooled from three independent experiments. *p* values were determined by two‐way ANOVA followed by Šídák's multiple comparisons test. (D) Nuclear volume analysis. Dots represent individual nuclei. n = 37 (stiff) and n = 91 (stiff+DNMTi) pooled from three independent experiments; *p* values were determined by an unpaired t‐test with Welch's correction. (E) ELISA quantification of IL‐6 and IL‐8 secretion from GFs (n = 3) cultured in soft or stiff hydrogels with or without DNMT inhibitor treatment; *p* values were determined by ordinary one‐way ANOVA followed by Tukey's multiple comparisons. Scale bars are as indicated. Data in C–E are presented as mean ± SD, and specific *p* values are indicated directly on the graphs where *p*<0.05 was considered statistically significant.

We hypothesized that matrix stiffness promotes differential levels of nuclear condensation to maintain distinct cellular phenotypes through DNA methylation pathways [23, 58, 59, 60, 61, 62, 63, 64] (Figure S5E). Fibroblasts in stiff gels generally exhibited higher DNMT1 nuclear expression (Figure 5A) and a more rounded nuclear shape that may represent a transcriptionally repressed state. GFs in both soft and stiff hydrogels were treated with a DNA methyltransferase inhibitor (DNMTi). We observed a dose‐dependent, significant increase in GF cellular spreading in stiff hydrogels treated with DNMTi (Figure 5B,C). DNMTi also caused a significant increase in nuclear volume for fibroblasts cultured in stiff hydrogels (Figure 5D), suggesting that DNA methylation contributes to the compact nuclear morphology observed in stiff ECM. This finding is consistent with previous studies showing that increased global DNA methylation is observed in stiffer matrices and that epigenetic processes integrate microenvironmental inputs to regulate fibroblast activation [7, 65, 66, 67]. The data strongly support our hypothesis that DNA methylation pathways regulate stiffness‐dependent responses [59, 60, 63]. Our finding that DNMTi can reverse the GF “stiff‐matrix phenotype” highlights the critical role of cellular epigenetic landscapes [64].

Further, we observed that DNMTi completely abrogated the immunomodulatory effect of matrix stiffness. GFs in stiff hydrogels treated with DNTMi now secreted significantly more IL‐6, achieving concentrations indistinguishable from those secreted by GFs in soft hydrogels (Figure 5E). Notably, this effect was selective, as IL‐8 secretion remained unchanged across all conditions, suggesting distinct mechanistic regulation for the two cytokines based on their promoter sensitivity to mechano‐epigenetic signals. We propose that the IL‐6 promoter is susceptible to mechanically induced DNA methylation. By inhibiting DNMTs, the epigenetic brake on IL‐6 is relieved, resulting in partial restoration of IL‐6 transcription when stimulated by TLR2. The suppression of IL‐8 may rely on a different mechanism that is resistant to pharmacological inhibition of DNMT and does not involve changes in CpG methylation status [59, 60, 62, 65]. We propose that matrix stiffness‐dependent DNA methylation acts as an epigenetic regulator of the pro‐inflammatory cytokine IL‐6 [53, 64, 65, 66, 67]. We propose that the soft matrix relieves this epigenetic control, allowing GFs to lose their intrinsic immunomodulatory response. DNA methylation and other matrix stiffness‐dependent mechanisms cooperate to fine‐tune the inflammatory setpoint [62]. Future studies are warranted to compare the binding and activity of known mechanosensitive transcription factors at the IL‐6 and IL‐8 promoters in stiff gels following DNMTi treatment.

### 2.11 Human Co‐Culture Model and Gingival Explant Model Support the Mechano‐Modulation of Inflammation in Periodontitis

**FIGURE 6:**
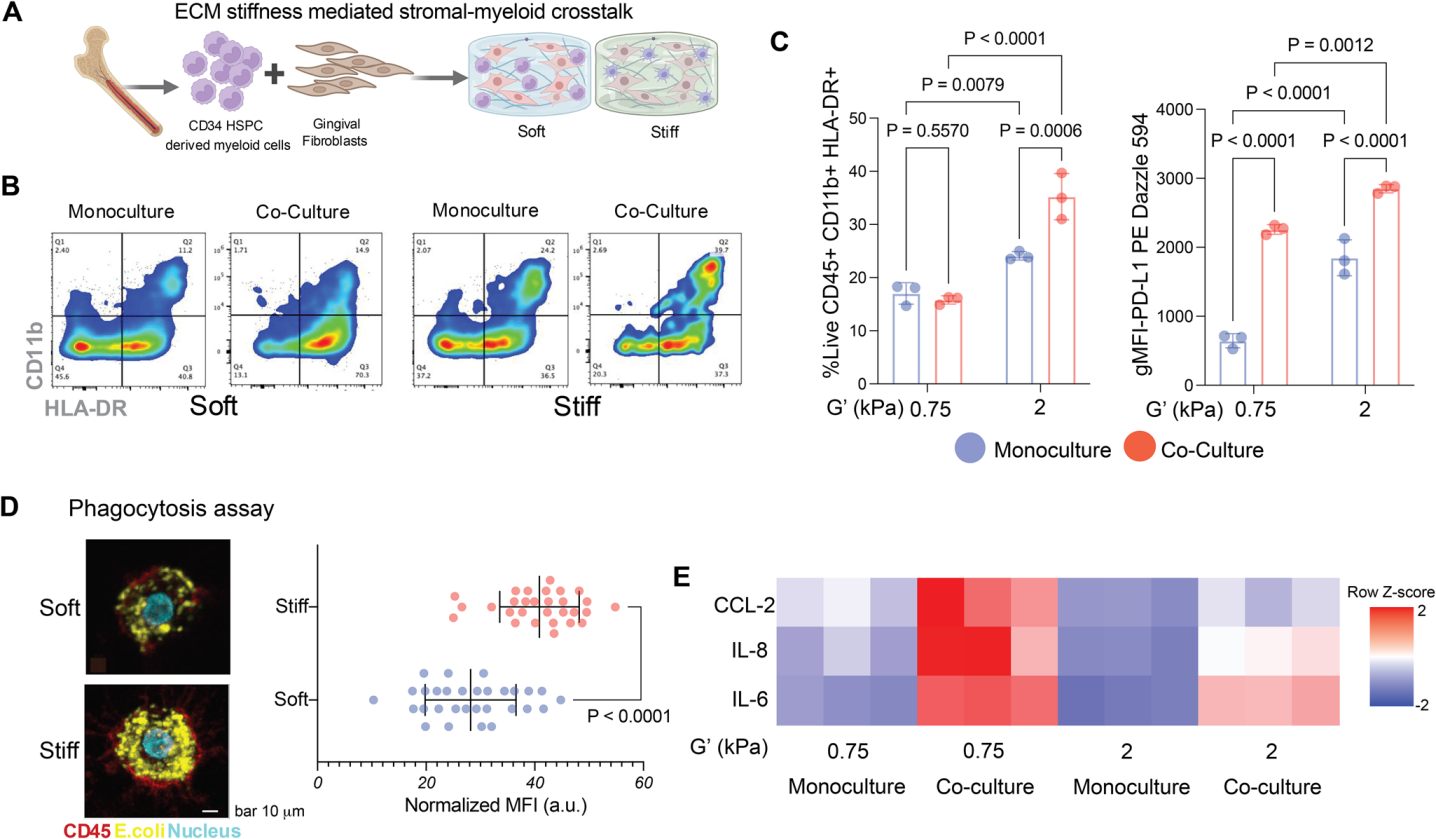
**ECM stiffness‐dependent stromal–myeloid crosstalk**. (A) Schematic depicting the 3D co‐culture of human CD34+ hematopoietic stem cell (HSC)‐derived myeloid precursors and GFs within soft (G′ 0.75 kPa) or stiff (G′ 2.0 kPa) hydrogels. Created with BioRender.com. (B) Representative flow cytometry plots and (C) quantification of myeloid cells (live, CD45+ gated) showing the percentage of CD11b+HLA‐DR+ cells (left) and PD‐L1 expression (right, gMFI). Data represent n = 3 biologically independent samples and are presented as mean ± SD. P‐values were determined by two‐way ANOVA followed by uncorrected Fisher's LSD. (D) Representative micrographs of soft and stiff ECM‐derived myeloid cells after incubation with pHrodo E. coli bioparticles, with quantification of phagocytosis by normalized MFI. Dots represent individual cells (n = 30 soft and n = 29 stiff) pooled from three independent experiments. Data are presented as mean ± SD. *p*‐values were determined by a two‐tailed unpaired t‐test. (E) Heatmap showing the relative secretion levels of IL‐6, IL‐8, and CCL2 in monocultures and co‐cultures (n = 3 biologically independent samples). Absolute cytokine concentrations (pg mL^−^^1^) were Z‐score normalized across samples, and visualized to highlight relative differences. *p*‐values represent group‐wise comparisons determined by two‐way ANOVA followed by Tukey's multiple comparisons. Scale bars are as indicated. For all panels, specific *p*‐values are indicated directly on the graphs, where *p*<0.05 was considered statistically significant.

We developed a clinically relevant model to examine the relationship between ECM mechanics and immune cell function. First, a mechanically tunable gingival ECM hydrogel mimicked stromal‐immune cell interactions by co‐culturing human GFs and CD34+ hematopoietic stem cell‐derived myeloid progenitors (Figure 6A). This strategy enables the evaluation of de novo differentiation of naïve myeloid cells and avoids the potentially confounding effects of previous antigen exposure of peripheral blood monocytes [61, 68, 69]. Naive myeloid cells were then encapsulated in either soft or stiff ECM hydrogels, either as monocultures or in co‐cultures with GFs. We then examined the differentiation, functional potential, and immunomodulatory phenotype of the stem cell‐derived myeloid cells to discern the integrated impact of fibroblast‐derived cues and mechanical signals. The combination of a stiff matrix and co‐culture with GF promoted the differentiation of naïve myeloid cells into functionally mature DCs, characterized by a higher population of HLA‐DR+ CD11b+ cells (∼40%) (Figure 6B). We investigated the immunomodulatory phenotype of these DCs by assessing PD‐L1 expression [70, 71]. Both matrix stiffness and GF co‐culture were potent inducers of PD‐L1. In immune cell monoculture, stiffness alone resulted in a notable increase in PD‐L1 expression. Yet co‐culture with GFs further upregulated both soft and stiff matrices (Figure 6B; Figure S6B). The highest PD‐L1 expression was observed in cells from the stiff co‐culture condition, indicating synergy between mechanical and paracrine signaling in determining the cells' regulatory potential. Furthermore, DCs differentiated in stiff hydrogels had a significantly greater phagocytic activity than those cultured in more compliant gels, as measured by the internalization of pH‐sensitive fluorescent E. coli particles (Figure 6D). These results suggest that a stiff environment, similar to healthy gingival tissue, supports the development of antigen‐presenting cells [48, 70, 71].

**FIGURE 7:**
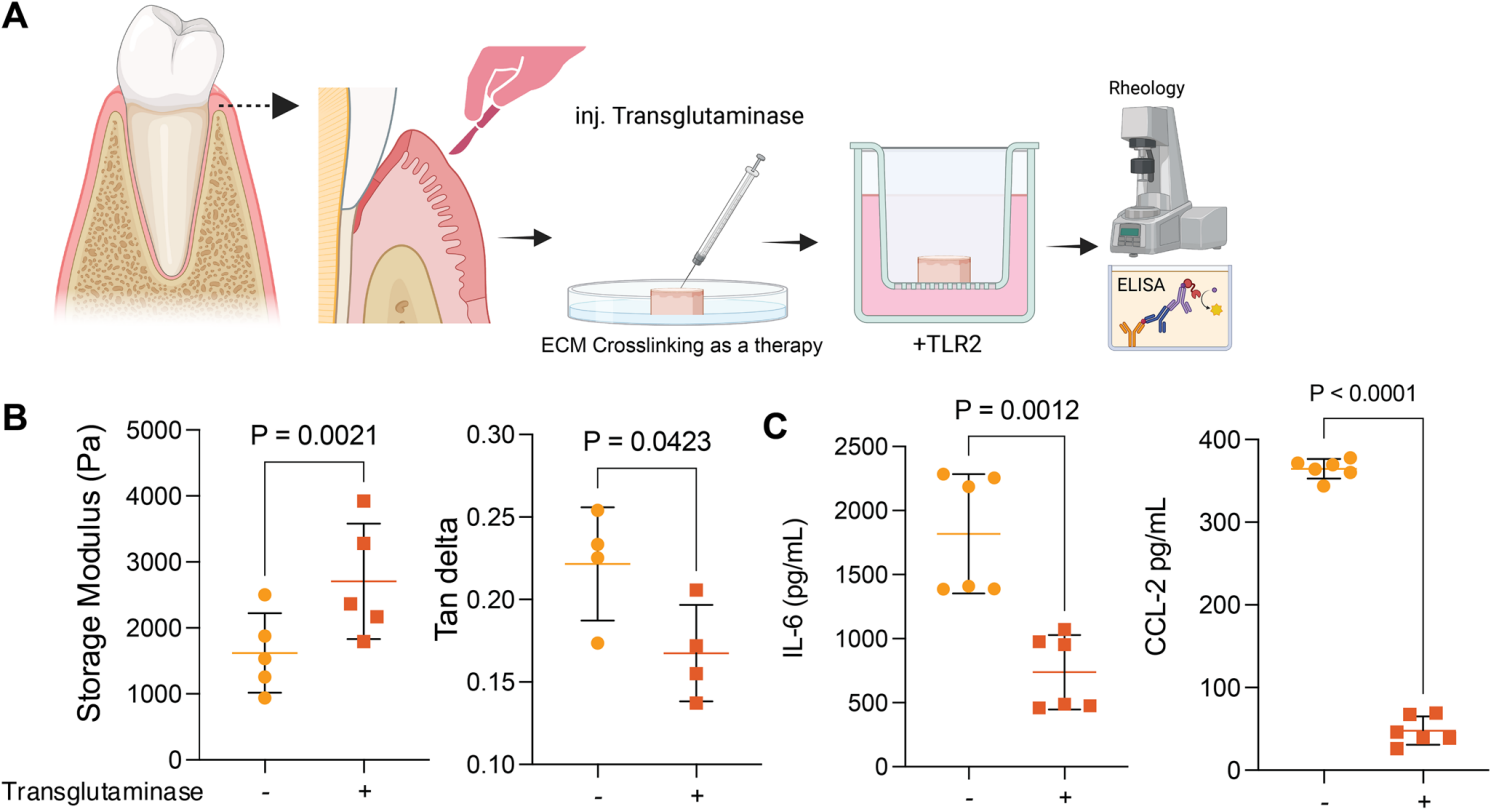
**Mechano‐modulation of periodontal inflammation via ECM stiffness**. (A) Schematic illustrating the experimental workflow for translational validation using an ex vivo human gingival explant model. The workflow involves transglutaminase injection to modulate ECM stiffness, followed by TLR2 stimulation and subsequent rheological and ELISA analysis. Created with BioRender.com. (B) Rheological characterization of human gingival tissue, showing the storage modulus (G′) and tan delta following transglutaminase treatment. *p*‐values were determined by a two‐tailed paired t‐test. (C) ELISA quantification of IL‐6 and CCL‐2 secretion from TLR2‐stimulated gingival explants. *p* values were determined by a two‐tailed paired t‐test. For B and C, data represent n = 3 biologically independent donors; dots indicate individual technical replicates (ex vivo explants) derived from these donors. Scale bars are as indicated. Data are presented as mean ± SD, and specific *p* values are indicated directly on the graphs, where *p*<0.05 was considered statistically significant.

To assess the combined influence of matrix stiffness and TLR2 signaling on the local inflammatory milieu, myeloid cell monocultures and gingival fibroblast co‐cultures within the respective soft and stiff hydrogels were challenged with TLR2, and the resulting cytokine profile was analyzed by ELISA. The assessment of the inflammatory response, summarized in the heatmap of Figure 7E, revealed a profound interplay between matrix mechanical properties and intercellular communication in regulating cytokine expression. The most significant pro‐inflammatory response was observed in the soft hydrogel (0.75 kPa) under co‐culture conditions. Here, the expression of key inflammatory cytokine (IL‐6) and chemokines, including CCL‐2 and IL‐8, was upregulated. This observation suggests that myeloid cells and GFs, when combined in a soft ECM environment, are highly inflammatory. Conversely, matrix stiffness, similar to that of healthy gingiva, suppressed the expression of all three measured cytokines (CCL‐2, IL‐8, and IL‐6), regardless of whether the cells were in monoculture or co‐culture status. This finding strongly suggests that ECM stiffness can dampen the inflammatory signals of myeloid‐fibroblast crosstalk. Overall, our data show that GFs in a stiff microenvironment induce myeloid precursors to become immunomodulatory phagocytic DCs with attenuated inflammatory responses, which are relevant for maintaining immune homeostasis in healthy gingival tissue [72, 73].

Next, we investigated whether enzymatic crosslinking of the gingival ECM with transglutaminase (Tg) can modulate the local immune response. Collagen has nonhelical ends (telopeptide regions), which are even more pronounced in degraded matrix, with accessible glutamine and lysine residues [25]. Tg acts on these sites, covalently linking the glutamine side chains to lysine via an acyl transfer reaction [74]. This causes crosslinking of adjacent collagen fibrils or collagen with adjacent ECM proteins like fibronectin and laminin [75, 76, 77]. We tested our hypothesis in an ex vivo human gingival explant model, which maintains the native tissue structure, cellular heterogeneity, and ECM composition (Figure 7A). This human tissue model eliminates inter‐species differences in animal models and provides clinically relevant data [15, 17]. Tg treatment successfully restored the mechanical properties of human gingival explant tissue. Rheological measurements showed that Tg‐treated tissue had a greater storage modulus (G′) and a lower tan delta (Tan δ = G′′/G′) value (Figure 7B,C). Further, when challenged with a TLR2 agonist to mimic a bacterial challenge, the Tg‐treated explants displayed a significant reduction in the release of key inflammatory mediators compared to non‐Tg controls (Figure 7C; Figure S7), including IL‐6 and CCL2. This result demonstrates that restoring the mechanical integrity of the gingival ECM impairs downstream inflammatory cascades. The lowering of CCL2 is especially relevant, as it would restrict the recruitment of further inflammatory monocytes, thereby disrupting a critical positive feedback loop that perpetuates chronic inflammation in periodontitis [78, 79].

Our data support ECM crosslinking as a potential therapeutic approach for periodontitis, as we demonstrate that restoring tissue stiffness is sufficient to shift the local immune environment from pro‐inflammatory to homeostatic and pro‐resolution. The translational potential of restoring healthy tissue stiffness is promising. However, oral tissues are colonized with complex bacterial biofilms [4]. Enhancing ECM cross‐linking cannot be viewed as a standalone therapy. ECM treatments would be adjunctive to standard non‐surgical mechanical debridement [9], which remains essential for reducing the dysbiotic microbial burden [3]. Future research will directly test this hypothesis by integrating polymicrobial biofilms into our stiffened hydrogel system to assess whether the mechanical checkpoint remains active and dominant in the presence of the primary etiological challenge. The insights derived from our work converge with recent advancements in translational biomaterials designed for periodontitis therapy. This finding is critical when evaluating clinically deployable hydrogel systems, such as the recently reported injectable short‐fiber hydrogel composed of nanocellulose (OBNC) and gelatin methacryloyl (GelMA) [80], hyaluronic acid‐based delivery systems, or adapting functional hydrogel technologies with antibacterial and remineralization properties [81, 82].

## 3 Conclusions, Limitations, and Outlook

**FIGURE 8:**
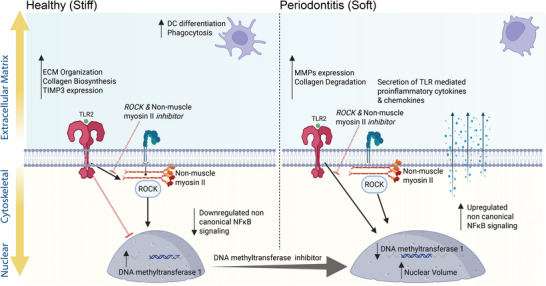
The ECM‐cytoskeletal‐nuclear axis regulates periodontal inflammation and fibroblast phenotype via DNMT1 and non‐canonical NFκB signaling. Created with BioRender.com.

This study establishes the mechanical microenvironment of the gingival connective tissue as a paramount and active regulator of GF phenotype and immune homeostasis. Through a tunable 3D gingival ECM‐mimicking hydrogel system, we identified a TLR2‐mediated checkpoint in which matrix stiffness controls GFs’ homeostatic and pathological responses. Stiff matrices, mimicking healthy tissue, drive a GF phenotype characterized by enhanced ECM synthesis, organized nuclear morphology, and robust suppression of pro‐inflammatory responses, primarily mediated by the non‐canonical NF‐κB pathway and influenced by DNA methylation. Conversely, soft, disease‐mimicking hydrogels promote a pro‐inflammatory, matrix‐degrading phenotype, recapitulating key transcriptional signatures observed in human periodontitis fibroblasts. We further elucidated that GFs in a stiff microenvironment promote the differentiation of immunomodulatory and phagocytic DCs Figure 8. In summary, our work provides compelling evidence for a paradigm shift in understanding periodontal disease pathogenesis, moving beyond traditional microbial models to highlight the central role of the physical microenvironment and opening new avenues for therapeutic intervention.

Native gingival ECM provides diverse biochemical cues beyond collagen Type I in our collagen‐alginate hydrogel. Our experimental and material design focused on isolating and controlling physical cues (stiffness and collagen architecture) as the dominant regulators of cellular mechanosensing. We achieved rheological properties that match those of healthy and diseased gingival tissue. Our system could incorporate decellularized ECM components in the future to provide a broader range of ligands. Although TLR2 stimulation simplifies the infectious milieu, future studies could validate the clinical generalizability of our findings using microbe‐specific stimuli, such as heat‐killed *F. nucleatum* or *P. gingivalis*, or by integrating polymicrobial periodontal biofilms. The insights gained from this study lay a robust foundation for several avenues of future research, including the link between DNA methylation and NFκB signaling, as well as the role of nuclear mechanics and chromatin remodeling in GFs. Future studies will also explore the heterogeneity of gingival fibroblasts using the 3D hydrogel model to identify novel therapeutic targets, followed by in vivo validation and the development of a bioengineered preclinical model [15] to translate these findings for the treatment of periodontitis Figure 8.

## Author Contributions

H.M. contributed to conceptualization, data curation, investigation, formal analysis, methodology, visualization, and writing, including the original draft and editing. N.T. and Y.C.C. were involved in investigation, methodology, and formal analysis. K.I.K. contributed to conceptualization, supervision, resources, and investigation. R.G.W. contributed to conceptualization, supervision, visualization, and writing. K.H.V. contributed to conceptualization, methodology, funding acquisition, supervision, visualization, writing, and resources. N.T. contributed to investigation, methodology, and formal analysis.

## Ethics Statement

All human gingival tissues and donor‐derived gingival fibroblasts (GFs) utilized in this research were obtained from the University of Pennsylvania Periodontology Clinic under Institutional Review Board oversight (IRB #844933; PI: Ko). These materials, which included deidentified human gingival biopsies from healthy or diseased donors, were used to establish ex vivo explant models or isolate primary GFs for 3D hydrogel systems. To maintain donor privacy, all human tissue samples were deidentified before being processed for experimental use. Furthermore, written informed consent was obtained from all participants prior to the collection and use of human tissues in accordance with ethical standards and institutional protocols.

## Conflicts of Interest

The authors declare no conflicts of interest.

## Supporting information




**Supporting File**: adma72486‐sup‐0001‐SuppMat.pdf.

## Data Availability

The data supporting the findings of this study will be openly available in Dyrad after publication (DOI: 10.5061/dryad.xksn02vw6).
