# Supplementary material for: Matrix Stiffness Governs Fibroblasts’ Regulation of Gingival Immune Homeostasis
Source: Adv Mater. 2026 Feb 8;38(15):e20717. doi: 10.1002/adma.202520717 (PMC12983442; doi:10.1002/adma.202520717)
Supplement: Supplementary file 1 — Supporting File: adma72486‐sup‐0001‐SuppMat.pdf. [file ADMA-38-e20717-s001.pdf]

Supporting Information

Matrix stiffness governs fibroblasts' regulation of gingival immune homeostasis

Authors: Hardik Makkar[1,7*], Nghi Tran[2], Yu-Chang Chen[3], Kang I Ko[4], Rebecca G. Wells[5,6], Kyle H. Vining[1,3,7$]

Affiliations:

[1] Center for Innovation & Precision Dentistry, University of Pennsylvania.

[2] Department of Bioengineering, School of Engineering and Applied Sciences, University of Pennsylvania

[3] Department of Materials Science and Engineering, School of Engineering and Applied Sciences, University of Pennsylvania

[4] Department of Periodontics, School of Dental Medicine, University of Pennsylvania

[5] Center for Engineering MechanoBiology, University of Pennsylvania

[6] Department of Medicine, Perelman School of Medicine, University of Pennsylvania

[7] Department of Preventive and Restorative Sciences, School of Dental Medicine, University of Pennsylvania

[$] Corresponding Author - viningk@upenn.edu

ORCiD

Hardik Makkar 0000-0002-8416-4138

Nghi Tran 0000–0002-3078-8199

Kang I Ko- 0000-0003-2967-8846

Yu-Chang Chen- 0009-0009-4678-3868

Rebecca G. Wells – 0000-0002-6988-4102

Kyle H. Vining – 0000-0002-4009-879X

Methods

Isolation of Donor-Derived Gingival Fibroblasts

Gingival fibroblasts were isolated from healthy human gingival tissue from the University of Pennsylvania, Periodontology Clinic (IRB exemption #844933, PI: Ko). Freshly obtained gingival tissues were washed in 1x HBSS without calcium and magnesium supplemented with antibiotic-antimycotic (1X, Gibco). The gingival tissues were cut into small pieces and placed in a T25 flask for 72 hours undisturbed and supplemented with Dulbecco's modified eagles medium (low glucose;1g/L) with 10% FBS and antibiotic-antimycotic (1X, Gibco). Cells were expanded, cryopreserved, and passages 2-4 were used for experiments.

G-CSF Mobilized CD34+ HSPC Cell Culture and Differentiation

Cells obtained from Fred Hutch Cancer Center were cultured at 20,000 cells/well 5 days per passage in serum-free StemSpan medium II (Stem Cell Technologies) supplemented with 100 ng/mL of stem cell factor (SCF; Peprotech), 100 ng/mL of Flt-3 ligand (FL3TL; Peprotech), 50 ng/mL of thrombopoietin (TPO; Peprotech), 20 ng/mL of IL-3 (Peprotech), and 700 nM UM729 (Stem Cell 15 Technologies). Cells were seeded into 96-well U-bottom plates with 200 µL of prewarmed media. The cultures were maintained at 37°C in a humidified atmosphere of 5% CO2. Half media changes were performed with freshly supplemented cytokines every 3 days. Expanded HSPCs at passage 2 (10 days total) were used for differentiation. Cells were differentiated for 7-days at a density of 1×10^5 cells/mL in Iscove's modified Dulbecco's medium (IMDM) containing 10% heat-inactivated FBS, 25 mmol/ L Hepes, 2 mmol/L L-glutamine, 0.1 mmol/L nonessential amino acids, 100 U/mL penicillin, and 100 µg/mL streptomycin (GIBCO), supplemented with 100 ng/mL of granulocyte-macrophage colony-stimulating factor (GM-CSF; Peprotech), 100 ng/mL of Flt-3 ligand (FL3TL; Peprotech) in 96 well U bottom plates (Costar, Cambridge, MA) at 37°C in a humidified atmosphere of 5% CO2. Media changes were performed every 3 days with freshly supplemented cytokines. Cells were cryopreserved in BamBanker (Fujifilm) at 7 days of myeloid differentiation.

Preparation of Gingival ECM hydrogels

Gingival ECM hydrogels were prepared as described previously[1]. Briefly, alginate (Pronova) was dissolved in a solution of 1x HBSS without calcium nor magnesium (14175095, Gibco) with HBSS/HEPES buffer) to a final w/v concentration of 5%. On the day of IPN fabrication, in a glass vial with a magnetic stir bar, bovine telo collagen I (Advanced Biomatrix) was neutralized on ice in v/v ratio of 17:3 with a solution created from 10x HEPES (14180046, ThermoFisher), 1 M HEPES, 1x HBSS, and 1M sodium hydroxide (NaOH) mixed in a v/v ratio of 10:2:2:1, respectively. Extra NaOH was added dropwise until the phenol red indicated a pH of ~7. HBSS/HEPES buffer was used to adjust the final concentration of collagen to 4 mg/mL. Subsequently, a 100 mg/mL CaCO3 slurry was prepared in endotoxin-free ultra-pure water. With these solutions ready, an aliquot of the CaCO3 slurry was incorporated into the neutralized collagen at a final concentration of 10, 20, or 30 mM. Then, alginate was added to the mixture at a final w/v concentration of 1%. These solutions were mixed with a magnetic stirrer. Next, a solution of 0.4 g/mL glucono-delta-lactone was formulated with HBSS/HEPES buffer immediately before its incorporation into the gel mixture at a 4x molar excess of CaCO3. The solution was stirred and mixed by pipetting. Working quickly, the liquid hydrogel solution was casted in 96 (60 μL) or 48 well-plates (150 μL) and incubated at 37°C for gelation and further experimentation.

Rheological Characterization of hydrogels and gingival tissues

The rheological properties of the gingival ECM hydrogels were measured on the HR-30 rheometer (TA Instrument) as shown previously[1d]. 100 μL of the hydrogel solution was dropped on top of the 20 mm Peltier plate at 37°C and loaded with a 20 mm parallel plate geometry. Excess solution was wiped off, and a solvent trap sealed with mineral oil was added to prevent dehydration. Time sweep was conducted at an oscillation of 1 Hz and strain of 2% until both storage modulus (G') and loss tangent (tan δ) reached an equilibrium (1 to 2 h), after which a frequency sweep from 0.1 to 10 Hz was performed under 2% strain. For the rheological measurements of human gingival tissues, samples were placed on the Peltier-controlled chamber at 37°C

submerged in HBSS/HEPES solution with 0.01N compressive preload. Viscoelastic properties such as storage modulus and tan(delta) are measured using a frequency sweep from 0.01-25 Hz with 0.5% strain, followed by a stress relaxation test with 10% strain over 5 seconds.

Cell culture and hydrogel encapsulation

To prevent collagen contraction, surface modification of 96 and 48 flat-bottom culture plates was done with a 2 mg/ml dopamine solution (Sigma Aldrich, USA) prepared in 10 mM Tris-HCl (pH 8.5) for 2h at room temperature[2]. After 2 hours of incubation, the dopamine solution was gently aspirated, and the surfaces were washed twice with sterile filtered deionized (DI) water to remove unbound dopamine. The washed substrates were kept sterile at room temperature until use. Gingival Fibroblasts (GFs) were isolated from healthy donor gingival tissues, cultured as explants. The isolated cells were cultured in Dulbecco's modified Eagle's medium (low glucose;1g/L) with 10% FBS. GFs suspended in HBSS/HEPES buffer and added to the collagen mixture, prior to the addition of alginate, at a final concentration of 1E06 cells/mL. Once all the hydrogel components were added and mixed, 60 μL or 150 μL hydrogel constructs were prepared in a 96- or 48-well flat-bottom well plates. The plates were then moved to a cell incubator at 37°C and 5% CO2. After 30 minutes, HBSS/HEPES buffer was added to the hydrogels, to maintain a pH ~7. Later, the buffer in the wells containing the ECM hydrogels was replaced with a low-serum defined media[3].

Exposure of gingival ECM hydrogels to pattern recognition receptor agonists

Gingival ECM hydrogels cultures for four days were exposed with TLR-2 agonist (Pam3CSK4, Invivogen), TLR-4 agonist (ultrapure *E. coli* LPS, Invivogen), TLR-3 agonist and TLR-9 agonist for 24 h. Following challenge, the culture supernatants were collected and stored at –80°C for downstream cytokine analysis. The concentrations are summarized in **Supplementary Table T2.**

Inhibition studies on Gingival ECM hydrogels

For pharmacological inhibition studies, the inhibitors were added to the culture media following encapsulation. The concentrations used for each inhibitor are summarized in **Supplementary Table T2.**

Retrieval of cells from ECM hydrogels and Viability assessment

Encapsulated cells were recovered by enzymatic digestion of the hydrogels[1a-c, 4]. Briefly, hydrogels were incubated with a digestion solution containing 300 U/mL collagenase type I (Thermo Scientific) and 34 U/mL alginate lyase (Sigma-Aldrich) in Dulbecco's phosphate-buffered saline (DPBS) supplemented with calcium and magnesium, and 0.5% bovine serum albumin (BSA), at 37°C for 15 minutes. An additional 60–150 μL of 300 U/mL collagenase I was then added with a second incubation at 37°C for 40 minutes. To terminate digestion, 0.5 mL of MACS buffer (DPBS without calcium and magnesium, supplemented with 2 mM EDTA and 0.5% BSA) was added, and the wells were washed using ice-cold PBS -/- and EasySep buffer (Stem Cell Technologies). Additional washes were performed if cells remain attached, or if undigested hydrogel was visible. The cell suspension was centrifuged at 400 × g for 5 minutes at 4°C for three times to obtain a clean cell pellet for downstream experiments.

Flow cytometry

Cells recovered from hydrogels were first stained with a fixable viability dye (Thermo Fisher Scientific) to exclude dead cells. Following viability staining, Fc receptors were blocked using human TruStain Fc receptor blocking reagent, and cells were stained with a panel of anti-human monoclonal antibodies targeting surface markers relevant to mesenchymal stromal cells, hematopoietic stem cell (HSC) expansion, myeloid differentiation, and immune activation (**Supplementary table T3)**. All antibodies were obtained from BioLegend. Staining was performed in eBioscience™ Flow Cytometry Staining Buffer (Thermo Fisher Scientific) according to the

manufacturer's instructions. After staining, cells were fixed with 2% paraformaldehyde for 20 minutes at room temperature, followed by three washes with FACs buffer. Fixed samples were stored at 4°C in the dark and analyzed within 48 hours. Single-color controls were prepared using compensation beads (BioLegend) for spectral unmixing. Data acquisition was performed using a 5-L Cytek® Aurora spectral flow cytometer at the Children's Hospital of Philadelphia (CHOP) Flow Cytometry Core. Data were analyzed using FCS Express.

Enzyme Linked Immunosorbent Assay (ELISA) for secretome analysis

In accordance with manufacturer's instructions, ELISAs for cytokines IL-6, IL-8, CCL-2 (all from Biolegend) were performed using the culture supernatants. The absolute cytokine values were normalized to the total protein content using BCA assay (Thermo Fisher Scientific).

Immunostaining of cryosections and whole mounts immunostaining.

The Gingival ECM hydrogels were fixed in 4% PFA (EMS), processed, and embedded in optimum cutting temperature compound (Tissue-Tek). Tissue sections (10μm thick). For immunostaining[5], the sections were first washed with DPBS, antigen retrieval with 0.5% Triton-X followed by blocking of nonspecific staining. The sections were incubated with respective primary antibodies overnight at 4°C, washed and incubated with respective secondary antibodies for 2 hours at 4°C (**Supplementary table T3**). The nuclei were counterstained with DAPI and mounted with anti-fade fluorescent mounting medium (Abcam). The slides were visualized under confocal microscope (Leica Stellaris, Leica Microsystems equipped with Leica Application Suite X software). Whole mount immunostaining was done as previously described[6]. Briefly, antigen retrieval and permeabilization was done by immersing the hydrogels in 0.5% Triton-X in phosphate-buffered saline (PBS) under orbital shaking for 2 h. The gels were then incubated in blocking solution (PBS containing 0.5% Triton-X, 10% goat serum, and 10% bovine serum albumin) overnight under orbital shaking. Tissues were incubated with primary antibodies (**Supplementary table T3**) for 2 d at 4 °C followed by washing under orbital shaking for 3 h. This was followed by incubation with respective secondary antibodies (**Supplementary table T3**) for 2 d at 4 °C and counterstaining with DAPI. Whole mounts were visualized using laser scanning confocal microscopy (Leica Stellaris, Leica Microsystems). Image analysis was done using Fiji (NIH) and Imaris software (Oxford Instruments).

Second Harmonic Generation Imaging and image analysis.

Second harmonic generation (SHG) imaging of formalin fixed paraffin embedded sections of human gingiva and viable whole gingival tissues were collected on Leica SP8-MP Upright with an external non-descanned hybrid detector (HyD – RLD2). The laser was tuned to 910nm, and the filter associated with the lens was 435~485nm. All images were processed from forward and backward SHG signals using z-stacks in FIJI (ImageJ) and Imaris software (Oxford Instruments). Briefly, to quantify the orientation of ECM collagen fibers, SHG images were analyzed using directionality plugin in FIJI/ImageJ[7]. This plugin computes a histogram that represents the data as a percentage of pixels in a given direction (between 1 to 180 degrees). The resultant direction of orientation and the pixel percentage counts are imported into Prism (Dotmatics). The pixel percentage counts for orientation of collagen fibers in a single layer are presented as a frequency histogram and that for the whole *z*-stack are presented as a heat map. Further, The CT-FIRE program (http://loci.wisc.edu/software/ctfire, version 1.3, beta 267) was utilized to quantify parameters of individual collagen fibers width, and straightness, in each SHG image.

Cell Viability analysis

The viability of the GFs in hydrogels was determined using Lactate Dehydrogenases (LDH) Assay (Promega) In accordance with manufacturer's instructions.

Phagocytosis Assay

Phagocytic activity was assessed using a fluorescent bioparticle uptake assay. Hydrogel retrieved cells were seeded at a density of 1×10^4 cells per well into glass bottom plates. Plates were incubated at 37 °C with 5% CO_2 for at least 1 hour to allow cells to settle. Fluorescently labeled bioparticles (e.g., pH-sensitive *E. coli* conjugates) were thawed, diluted 20X and 100 μL of the prepared bioparticle suspension was added. Cells were then incubated for 90 minutes at 37 °C to allow for phagocytosis and intracellular acidification. The plates were imaged using confocal microscope (Leica Stellaris, Leica Microsystems) and image analysis was done using Fiji (NIH).

Bulk RNA- sequencing and analysis

Cells encapsulated in hydrogels were harvested and lysed using RLT buffer supplemented with 1% β-mercaptoethanol (β-ME) (Sigma-Aldrich), following standard protocols for disruption and homogenization of cell pellets. Briefly, cell pellets were resuspended in 350 μL of RLT + β-ME and pipetted vigorously to ensure complete lysis and homogenization. Lysates were stored at –80°C or processed immediately for RNA extraction. Total RNA was isolated using a commercial column-based purification kit (RNeasy Mini Kit, Qiagen), according to the manufacturer's instructions. This included an on-column DNase I digestion step to remove genomic DNA contamination and elution in RNase-free water. RNA concentration and integrity were assessed by spectrophotometry (NanoDrop) prior to sequencing. Bulk RNA sequencing was performed by Azenta Life Sciences. Library preparation, quality control, sequencing, and initial data processing were conducted by Azenta. Differential gene expression results from bulk RNA sequencing were analyzed using Reactome pathway enrichment to identify significantly altered biological pathways. Enrichment analysis was performed using the Reactome Pathway Database (https://reactome.org) by submitting the list of significantly upregulated and downregulated genes. Overrepresentation analysis was carried out using the Reactome analysis tool, which applies a hypergeometric test to assess pathway enrichment, followed by false discovery rate (FDR) correction for multiple testing. Pathways with an FDR < 0.05 were considered significantly enriched. The top enriched up- and downregulated pathways were visualized based on normalized enrichment scores and p-values to highlight key biological processes altered in the dataset. For a dedicated analysis of gene sets, z-score normalized gene counts were plotted as heatmaps. In parallel, Gene Ontology (GO) enrichment analysis was performed using the NIH DAVID Bioinformatics Resources (v6.8). Official gene symbols were uploaded, and functional annotation clustering was conducted. Enrichment significance was assessed using DAVID's modified Fisher Exact test (EASE score), with multiple testing correction by the Benjamini–Hochberg method. GO biological process terms with adjusted $p < 0.05$ were considered significantly enriched.

Human gingival explants culture and transglutaminase injection

Deidentified human gingival tissues were obtained from the University of Pennsylvania Periodontology Clinic (IRB #844933, PI: Ko). Immediately following collection, explants were washed with HBSS buffer (without calcium and magnesium) supplemented with 1% antibiotic–antimycotic solution. Transglutaminase (10 U; SAE0159, Sigma) reconstituted in PBS was injected into the tissues using a hypodermic syringe; control samples received PBS alone. Tissue punches (2 mm) were prepared using a sterile biopsy punch and placed in transwell inserts under air–liquid interface conditions. Explants were cultured for 3 days in low-serum medium containing ascorbic acid (10 mg ml–1), hydrocortisone (50 μg ml–1), basic fibroblast growth factor (10 ng ml–1), vascular endothelial growth factor (20 ng ml–1), epidermal growth factor (5 ng ml–1), 1% penicillin–streptomycin, and SITE supplement (selenium, 5 ng ml–1; insulin, 10 μg ml–1; transferrin, 5.5 μg ml–1; ethanolamine, 2 μg ml–1). During the final 24 h of culture, explants were treated with a TLR2 agonist (Pam3CSK4, 1 μg ml–1). Following culture, tissues were subjected to rheological measurements, and conditioned media were collected for ELISA analysis.

Statistical Analysis

Data are presented as mean ± standard deviation (SD) from at least three biologically independent donors or experiments. For all datasets, the validity of assumptions was confirmed, and all data passed normality testing prior to parametric analysis. Significant differences between two groups were determined using two-tailed unpaired or paired t-tests, including Welch's correction for unequal variances where appropriate. For comparisons involving more than two groups, ordinary one-way or two-way ANOVA was performed. Following

ANOVA, specific post-hoc tests were applied as indicated: Tukey's, Dunnett's, or Šídák's multiple comparisons tests, the original FDR method of Benjamini and Hochberg, or uncorrected Fisher's LSD. A P-value of < 0.05 was considered statistically significant. All statistical analyses and graphing were performed using Prism (Dotmatics). Additional data processing was conducted using FCS Express or Flow Jo for flow cytometry, Fiji (NIH) and Imaris (Oxford Instruments) for image analysis, and Reactome or NIH DAVID for bioinformatics analysis.

Supplementary Figures

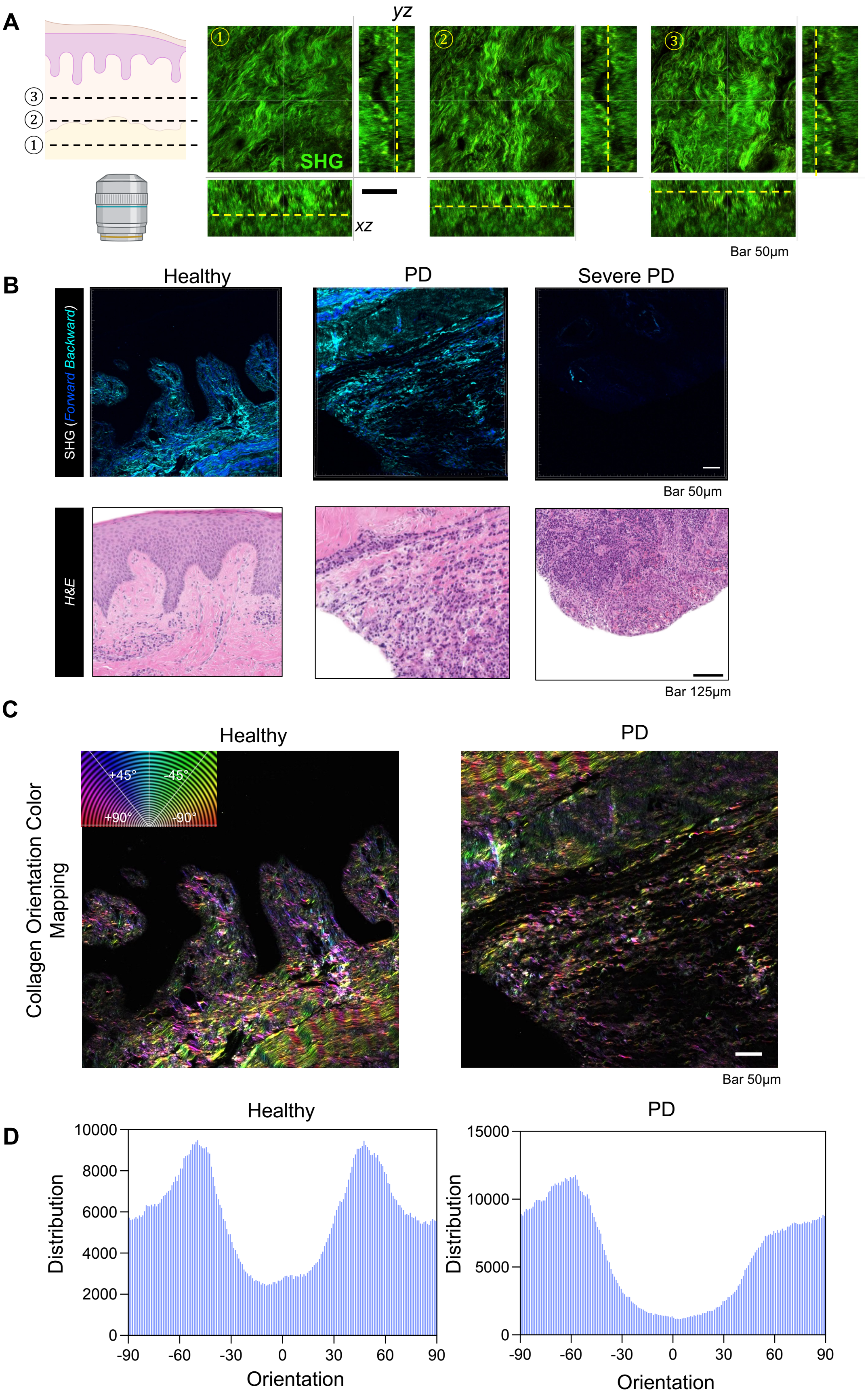

Supplementary Figure S1. (A) z-sections of whole-mount SHG image of gingival connective tissue. **(B)** Comparison of tissue morphology and collagen structure across healthy, periodontitis (PD), and severe periodontitis (Severe PD) samples. The Top Row features Second Harmonic Generation (SHG) microscopy images, with the signal often color-coded for forward and backward scattering. The Bottom Row shows corresponding Hematoxylin and Eosin (H&E) stained images. Both SHG and H&E images are taken from the same tissue region, but are from adjacent sections, where the physical sectioning process results in slight morphological differences between the optical (SHG) and physical (H&E) views. **(C)** Collagen orientation color map of collagen fibers in healthy and diseased gingival connective tissue. **(D)** The angular distribution histogram for the healthy and diseased site (PD). Scale bars are as indicated.

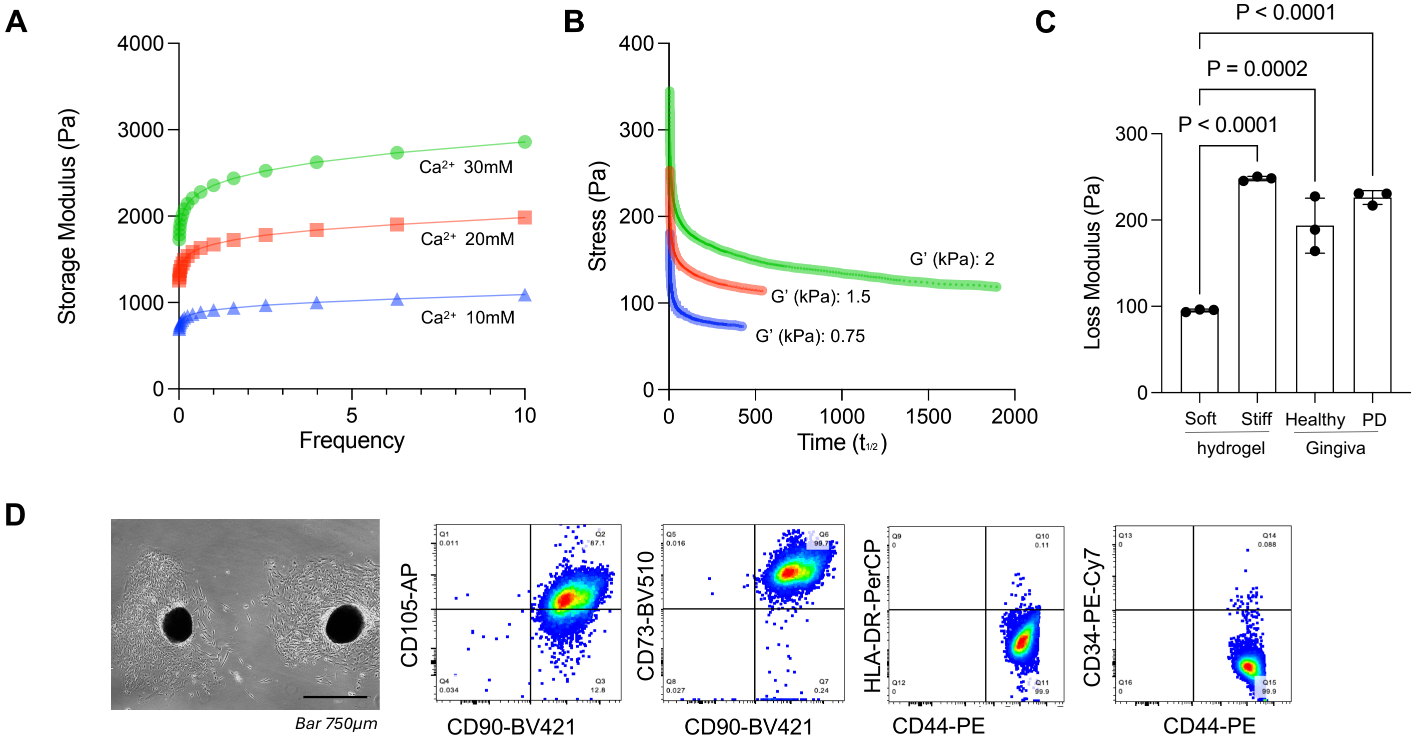

Supplementary Figure S2. (A) Oscillatory shear rheology- frequency sweep and **(B)** stress relaxation behavior of gingival ECM hydrogels **(C)** Loss modulus of gingival ECM hydrogels, healthy gingiva and gingival tissue with periodontal disease (PD). For rheology measurements, n = 3 biologically independent donors per group. **(D)** Isolation of GFs from healthy donors and culture as explants. Flow cytometry confirms the mesenchymal stromal surface marker profile, showing cells are positive for CD105, CD73, and CD90, and negative for hematopoietic and endothelial markers HLA-DR, CD44, and CD34. Scale bars are as indicated. Data are shown as mean ± SD. P-values from statistical tests are indicated.

A

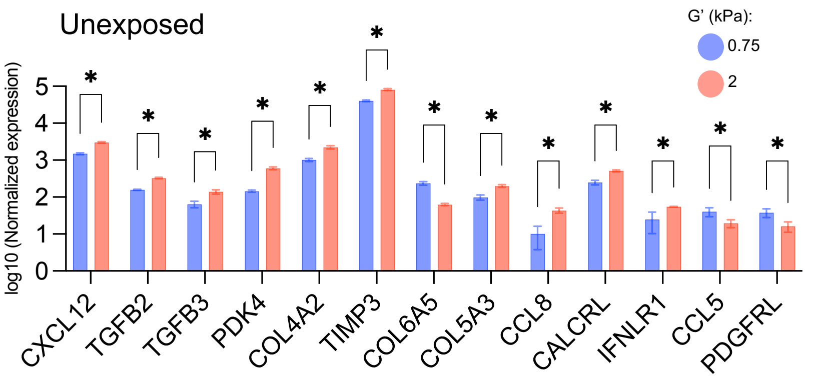

Gene	P value	q value
CXCL12	0.000101	0.000286
TGFB2	0.000025	0.000170
TGFB3	0.006385	0.009684
PDK4	0.000025	0.000170
COL4A2	0.000521	0.001185
TIMP3	0.000105	0.000286
COL6A5	0.000067	0.000286
COL5A3	0.002407	0.004107
CCL8	0.029657	0.033734
CALCRL	0.001482	0.002890
IFNLR1	0.047410	0.049780
CCL5	0.025458	0.031592
PDGFRL	0.019543	0.026677

B

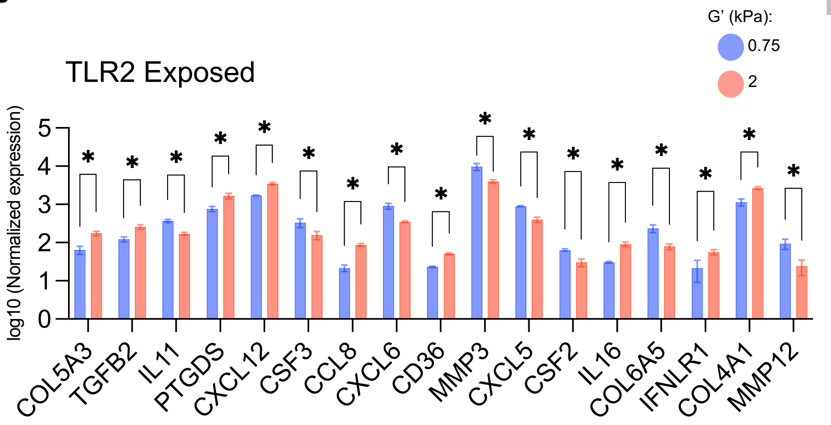

Gene	P value	q value
COL5A3	0.002093	0.004584
TGFB2	0.003592	0.005829
IL11	0.000690	0.002462
PTGDS	0.006381	0.009492
CXCL12	0.000244	0.001088
CSF3	0.031890	0.033484
CCL8	0.000175	0.001088
CXCL6	0.003432	0.005829
CD36	0.000062	0.001088
MMP3	0.007897	0.010069
CXCL5	0.000234	0.001088
CSF2	0.002311	0.004584
IL16	0.001201	0.003063

C

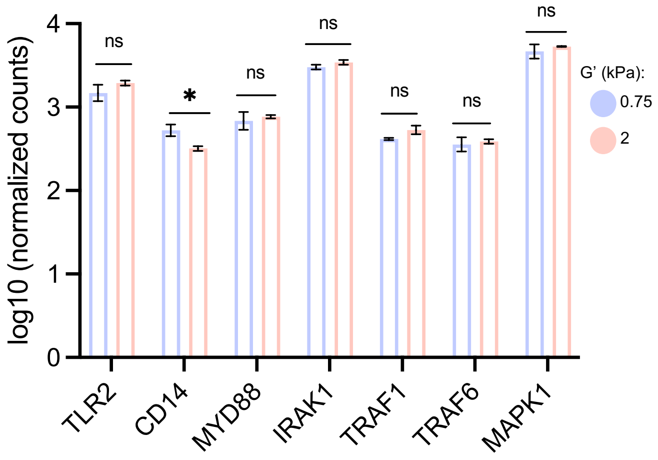

Supplementary Figure S3. Normalized expression of genes in gingival fibroblasts encapsulated in soft and stiff ECM hydrogels without **(A)** and with **(B)** TLR2 agonist. All data are shown as mean ± SD. P-values and q values from statistical tests are indicated in tables. **(C)** Gene expression analysis of key components of the TLR2 signaling pathway in gingival fibroblasts (GFs) cultured in soft versus stiff hydrogels. Data are shown as mean ± SD (n=3). P-values from statistical tests are indicated.

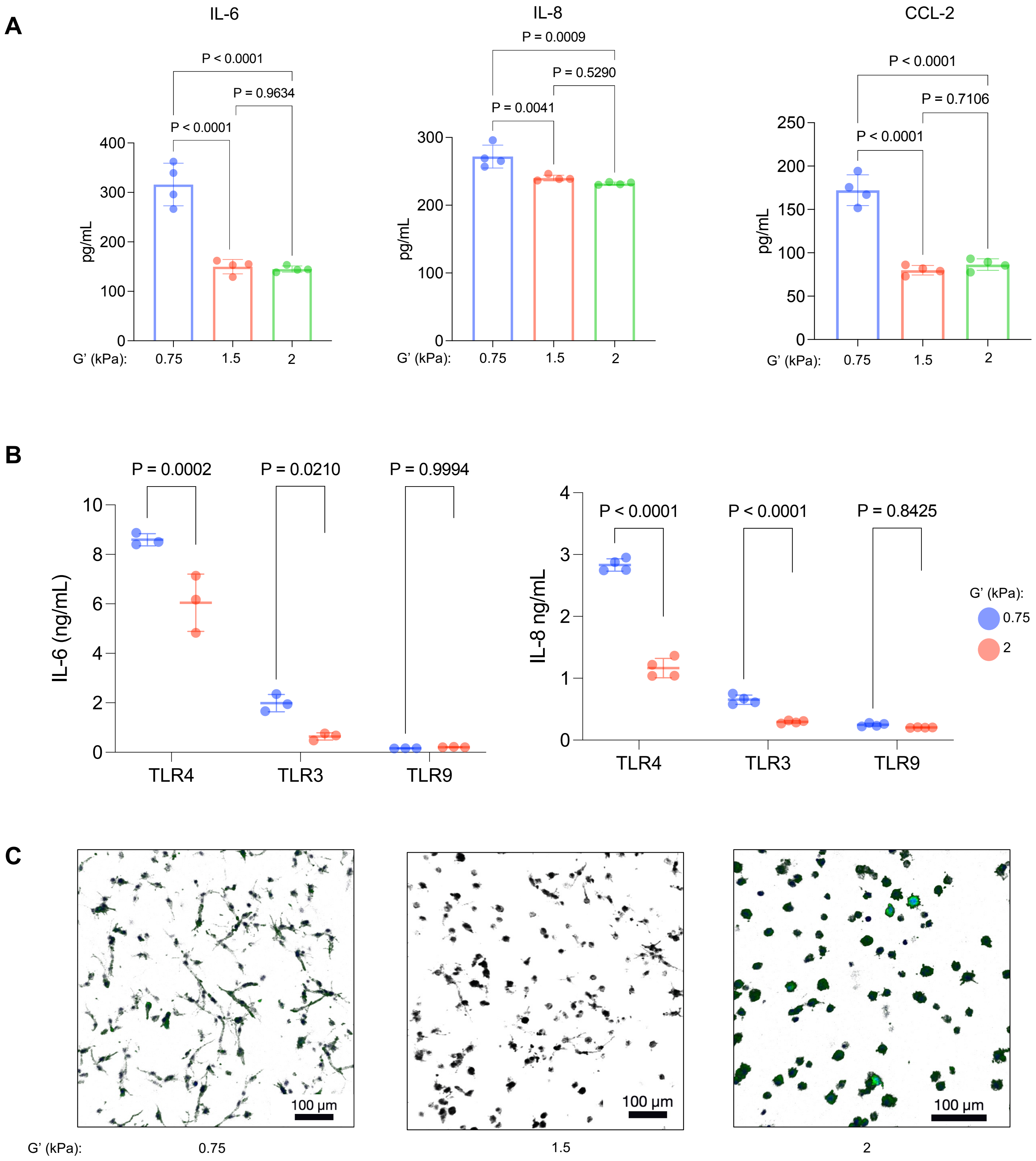

Supplementary Figure S4. **(A)** ELISA quantification of secreted IL-6, IL-8, and CCL-2 from GFs cultured in hydrogels of varying stiffness. **(B)** Secretion of IL-6 and IL-8 by GFs encapsulated in soft and stiff hydrogels exposed with TLR3, TLR4 & TLR9 agonists. Scale bars are as indicated. **(C)** Representative confocal immunofluorescence images showing GFs cultured on hydrogels with increasing stiffness (G′: 0.75, 1.5, and 2.0kPa). Scale bars are as indicated. Data are shown as mean ± SD. P-values from statistical tests are indicated.

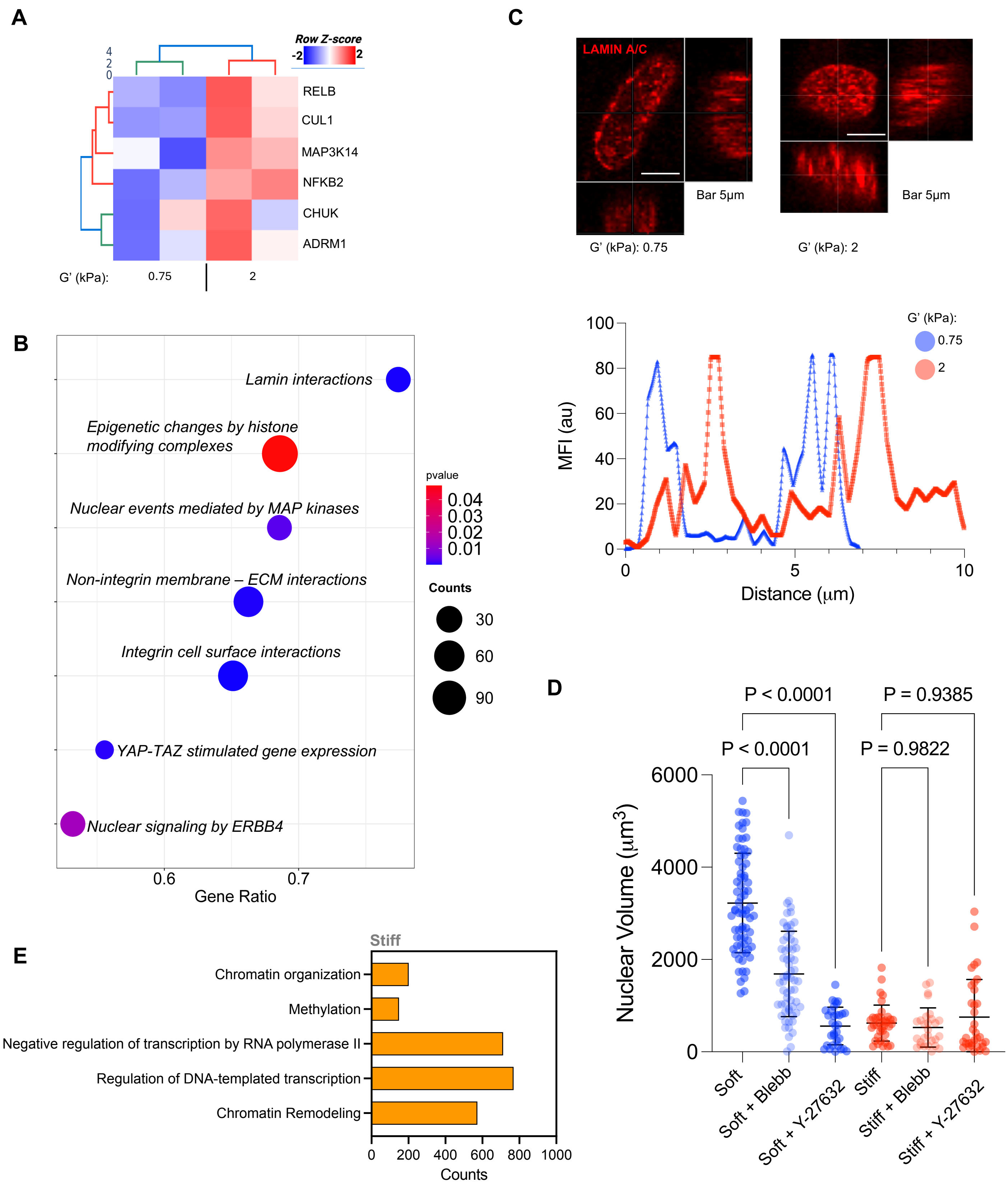
A
Row Z-score
-2
2
4
2
0
RELB
CUL1
MAP3K14
NFKB2
CHUK
ADRM1
G' (kPa):
0.75
2
B
Lamin interactions
Epigenetic changes by histone modifying complexes
Nuclear events mediated by MAP kinases
Non-integrin membrane – ECM interactions
Integrin cell surface interactions
YAP-TAZ stimulated gene expression
Nuclear signaling by ERBB4
0.6
0.7
Gene Ratio
pvalue
0.04
0.03
0.02
0.01
Counts
30
60
90
C
LAMIN A/C
Bar 5µm
Bar 5µm
G' (kPa): 0.75
G' (kPa): 2
MFI (au)
Distance (µm)
G' (kPa):
0.75
2
0
20
40
60
80
100
0
5
10
D
Nuclear Volume (µm³)
P < 0.0001
P < 0.0001
P = 0.9385
P = 0.9822
0
2000
4000
6000
Soft
Soft + Blebb
Soft + Y-27632
Stiff
Stiff + Blebb
Stiff + Y-27632
E
Stiff
Chromatin organization
Methylation
Negative regulation of transcription by RNA polymerase II
Regulation of DNA-templated transcription
Chromatin Remodeling
0
200
400
600
800
1000
Counts

Supplementary Figure S5. **(A)** Heatmap of differentially expressed genes in non-canonical NF-κB pathway in GFs cultured in soft and stiff hydrogels. **(B)** A bubble plot illustrates the enrichment of pathways associated with nuclear and epigenetic changes in GFs cultured in stiff (2kPa) hydrogels. **(C)** Immunofluorescence images show the distribution of Lamin A/C (red) and the cell nucleus (stained with DAPI, blue) in cells on soft and stiff substrates. The line scan profile below the images quantifies the fluorescence intensity of Lamin A/C across the nucleus, showing a more peripheral localization of lamin AC in GFs encapsulated in stiff hydrogels compared to more diffuse expression under soft condition. **(D)** Plots representing the nuclear volume of GFs in soft and stiff hydrogels treated with myosin II inhibitor Blebbistatin or the ROCK inhibitor Y-27632. **(E)** Plot showing the enrichment of Gene Ontology (GO) terms related to epigenetic regulation of GFs in stiff hydrogels. Scale bars are as indicated. Data are shown as mean ± SD. P-values from statistical tests are indicated.

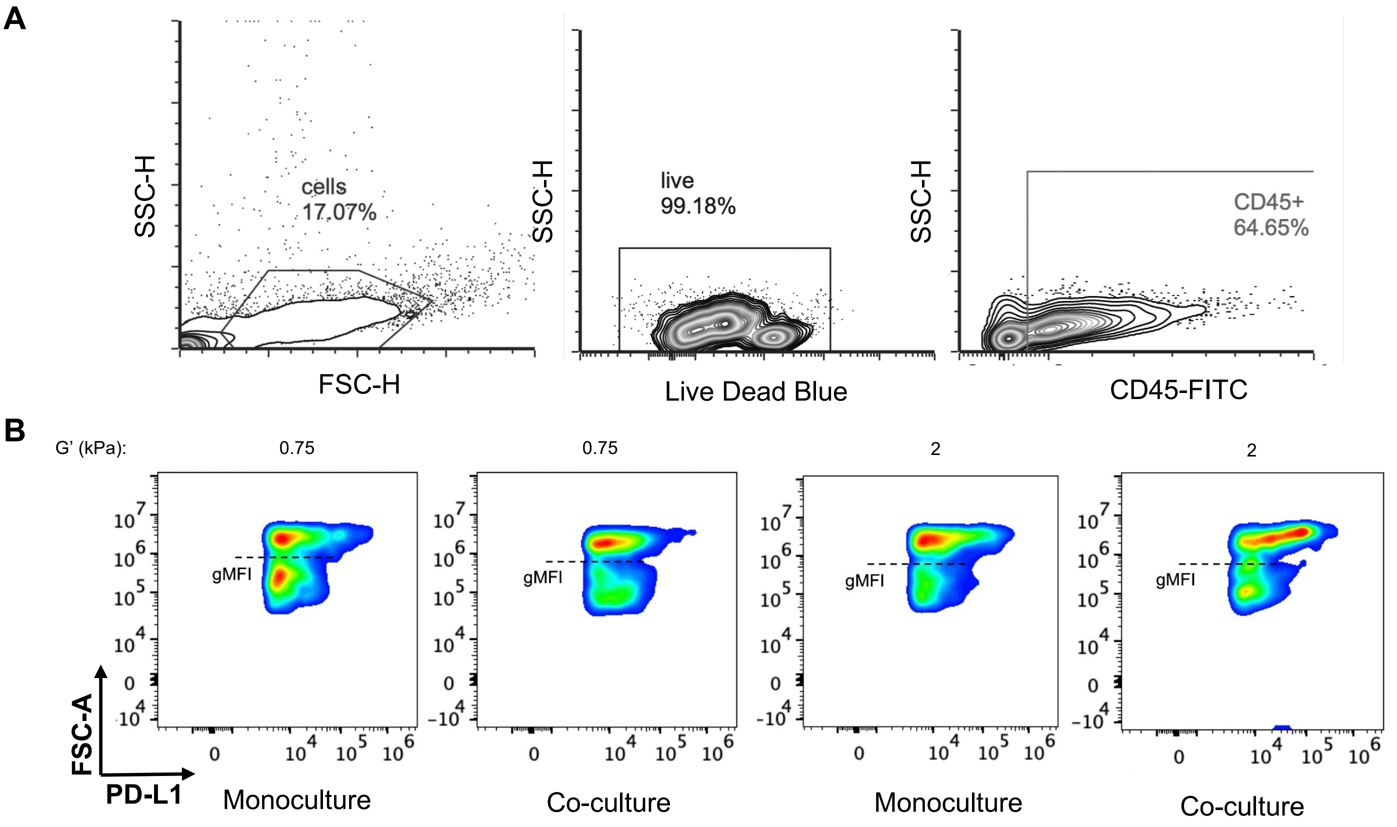

Supplementary Figure S6. (A) Gating strategy for myeloid cells and gingival fibroblasts co-cultured in gingival ECM hydrogels. **(B)** Representative flow cytometry plots showing the expression of PD-L1 on myeloid cells (gated on live, CD45+ cells) after culture in soft or stiff ECM hydrogels, either in monoculture or in co-culture with gingival FBs. Data are shown as mean ± SD. P-values from statistical tests are indicated.

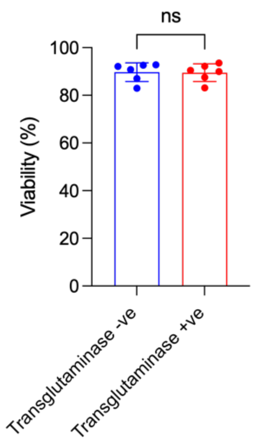

Supplementary Figure S7. Viability of gingival explants ex vivo with and without transglutaminase treatment. All data are shown as mean ± SD. P-values from statistical tests are indicated

Supplementary Table T1. Formulations of artificial ECM hydrogels.

Hydrogel	Collagen (mg/mL)	VLVG alginate (% w/v)	$CaCO_3$ (% w/v)	GDL (mM)
**Soft viscous**	4	1	0.10	40
**Stiff viscous**	4	1	0.30	120

Supplementary Table T2. Pharmacological inhibitors and cytokine treatments.

Name	Synonyms	Target	Concentration	Manufacturer
Pam3CSK4	TLR2 ligand	TLR2	1 µM	Invivogen
Poly(I:C)HMW	TLR3 ligand	TLR3	10 ug/mL	Invivogen
ODN-M362	TLR9 ligand	TLR9	5 µM	Invivogen
IKK inhibitor	BMS-345541	IKK	10 µM	Selleckchem
Decitabine	DNMT inhibitor	DNMT	0.5, 1 µM	Selleckchem
Blebbistatin		NMII	25 µM	Selleckchem
ROCK inhibitor	Y-27632	ROCK	25 µM	Selleckchem
LPS	LPS	TLR4 agonist	1 ug/mL	Invivogen
IFN-gamma	IFNgamma	activates TNFalpha	100 ng/mL	Peprotech

Supplementary Table T3. Details of primary and secondary antibodies used in the study

Antibody	Specification	Dilution	Source
Phalloidin i-Fluor		1:1000	Abcam (ab176753)
Lamin A/C (4C11)	Mouse monoclonal	1:400	Cell Signaling Technologies (4777T)
Phospho RelB (Ser552)	Rabbit monoclonal	1:200	Cell Signaling Technologies (4999S)
DNMT1 (D63A6)	Rabbit monoclonal	1:200	Cell Signaling Technologies(5032T)
Secondary antibody	Alexa Fluor-488 (Goat anti-mouse)	1:250	Molecular Probes (#A11001)
Secondary antibody	Alexa Fluor-594 (Goat anti-rabbit)	1:300	Molecular Probes (#A11037)
Brilliant Violet 421™ anti-human CD90	Mouse monoclonal	1:100	Biolegend (328121)
APC anti-human CD105	Mouse monoclonal	1:20	Biolegend (323207)
PerCP anti-human HLA-DR	Mouse monoclonal	1:100	Biolegend (307627)
PE/Cyanine 7 anti-human CD34	Mouse monoclonal	1:100	Biolegend (343515)
PE anti-human CD44	Mouse monoclonal	1:100	Biolegend (397503)
Brilliant Violet 421™ anti-human CD73	Mouse monoclonal	1:100	Biolegend (344043)
PE/Dazzle™ 594 anti-human PD-L1	Mouse monoclonal	1:100	Biolegend (329731)
Brilliant Violet 711™ anti-human CD11b	Mouse monoclonal	1:100	Biolegend (301343)

Supplementary Table T4. Statistical comparison of cytokine levels measured by ELISA for main figure 6E. P-values represent group-wise comparisons of IL-6, IL-8, and CCL2 concentrations as determined by ELISA.

IL-6	Adjusted P Value
Soft Monoculture Vs. Soft Co-Culture	<0.0001
Soft Monoculture Vs. Stiff Monoculture	0.1895
Soft Monoculture Vs. Stiff Co-Culture	<0.0001
Soft Co-Culture Vs. Stiff Monoculture	<0.0001
Soft Co-Culture Vs. Stiff Co-Culture	0.0008
Stiff Monoculture Vs. Stiff Co-Culture	<0.0001

IL-8	Adjusted P Value
Soft Monoculture Vs. Soft Co-Culture	0.0025
Soft Monoculture Vs. Stiff Monoculture	0.7784
Soft Monoculture Vs. Stiff Co-Culture	0.1962

Soft Co-Culture Vs. Stiff Monoculture	0.0012
Soft Co-Culture Vs. Stiff Co-Culture	0.0214
Stiff Monoculture Vs. Stiff Co-Culture	0.0624

**CCL-2**	**Adjusted P Value**
Soft Monoculture Vs. Soft Co-Culture	0.0083
Soft Monoculture Vs. Stiff Monoculture	0.5085
Soft Monoculture Vs. Stiff Co-Culture	0.9974
Soft Co-Culture Vs. Stiff Monoculture	0.0023
Soft Co-Culture Vs. Stiff Co-Culture	0.007
Stiff Monoculture Vs. Stiff Co-Culture	0.6056

Supplementary Table T5 – Statistical tests in data of main figure

**Data**	**Test**	**Passed Normality test?**
**Fig. 1**I	Ordinary one-way ANOVA<br>Dunnett's multiple comparisons test	Yes
**Fig.** 2D	Ordinary one-way ANOVA<br>Dunnett's multiple comparisons test	Yes
**Fig.** 3C	Ordinary one-way ANOVA<br>Tukey's multiple comparisons	Yes
**Fig.** 4B	Unpaired t test, two-tailed	Yes
**Fig.** 4D	Two-way ANOVA<br>Šídák's multiple comparisons test	Yes
**Fig.** 4E	Two-way ANOVA<br>Original FDR method of Benjamini and Hochberg	Yes
**Fig.** 4H	Unpaired t test, two-tailed	Yes
**Fig.** 4I	Two-way ANOVA<br>Šídák's multiple comparisons test	Yes
**Fig.** 5C	Two-way ANOVA<br>Šídák's multiple comparisons test	Yes
**Fig.** 5D	Unpaired t test with Welch's correction	Yes
**Fig.** 5E	Ordinary one-way ANOVA<br>Tukey's multiple comparisons	Yes

**Fig.** 6C	Two-way ANOVA<br>Uncorrected Fisher's LSD	Yes
**Fig.** 6D	Unpaired t test, two-tailed	Yes
**Fig.** 6E	Two-way ANOVA<br>Tukey's multiple comparisons	
**Fig.** 7B	Paired t test, two-tailed	Yes
**Fig.** 7C	Paired t test, two-tailed	Yes

AUTHENTICATION OF KEY BIOLOGICAL AND/OR CHEMICAL RESOURCES

1) All acquired compounds and reagents were authenticated for both identity and purity, based on certificate of analysis from manufacturers.
2) The fluorescent probes, laser and filter parameters related to the confocal microscopy have been extensively tested and selected to minimize potential cross talk between different excitation and emission channels
3) Primary human cells for in vitro experimentation were routinely tested for mycoplasma and bacterial contaminations.

References

[1] a)J. J. Lim, K. H. Vining, D. J. Mooney, B. J. Blencowe, *Proc Natl Acad Sci U S A* **2024**, 121, e2404146121; b)K. H. Vining, A. E. Marneth, K. Adu-Berchie, J. M. Grolman, C. M. Tringides, Y. Liu, W. J. Wong, O. Pozdnyakova, M. Severgnini, A. Stafford, G. N. Duda, F. S. Hodi, A. Mullally, K. W. Wucherpfennig, D. J. Mooney, *Nat Mater* **2022**, 21, 939; c)K. H. Vining, A. Stafford, D. J. Mooney, *Biomaterials* **2019**, 188, 187; d)K. Zhang, Z. Li, Y. C. Chen, I. C. Yoon, A. Graham, K. Vining, *ACS Appl Bio Mater* **2025**, 8, 3899.

[2] S. E. Park, A. Georgescu, J. M. Oh, K. W. Kwon, D. Huh, *ACS Appl Mater Interfaces* **2019**, 11, 23919.

[3] H. Makkar, Y. Zhou, K. S. Tan, C. T. Lim, G. Sriram, *Adv Healthc Mater* **2023**, 12, e2202376.

[4] K. Adu-Berchie, Y. Liu, D. K. Y. Zhang, B. R. Freedman, J. M. Brockman, K. H. Vining, B. A. Nerger, A. Garmilla, D. J. Mooney, *Nat Biomed Eng* **2023**, 7, 1374.

[5] H. Makkar, S. Atkuru, Y. L. Tang, T. Sethi, C. T. Lim, K. S. Tan, G. Sriram, *J Tissue Eng* **2022**, 13, 20417314221111650.

[6] H. Makkar, C. T. Lim, K. S. Tan, G. Sriram, *Biofabrication* **2023**, 15.

[7] a)Y. Luo, H. Makkar, Y. Hu, K. Chen, P. K. Purohit, K. H. Vining, *ACS Materials Letters* **2025**; b)S. Atkuru, G. Muniraj, T. Sudhaharan, K. H. Chiam, G. D. Wright, G. Sriram, *J Periodontal Res* **2021**, 56, 108.
